# Global distribution, host range and prevalence of *Trypanosoma vivax*: a systematic review and meta-analysis

**DOI:** 10.1186/s13071-021-04584-x

**Published:** 2021-01-25

**Authors:** Eyerusalem Fetene, Samson Leta, Fikru Regassa, Philippe Büscher

**Affiliations:** 1 College of Veterinary Medicine and Agriculture, Addis Ababa University, P. O. Box 34, Bishoftu, Ethiopia; 2 FDRE Ministry of Agriculture, P.O.Box 62347/3735, Addia Ababa, Ethiopia; 3 Institute of Tropical Medicine, Department of Biomedical Sciences, Nationalestraat 155, 2000 Antwerp, Belgium

**Keywords:** *Trypanosoma vivax*, Global distribution, Host species, Meta-analysis, Pooled prevalence, Domestic animals, Wild fauna

## Abstract

**Background:**

Trypanosomosis caused by *Trypanosoma vivax* is one of the diseases threatening the health and productivity of livestock in Africa and Latin America. *Trypanosoma vivax* is mainly transmitted by tsetse flies; however, the parasite has also acquired the ability to be transmitted mechanically by hematophagous dipterans. Understanding its distribution, host range and prevalence is a key step in local and global efforts to control the disease.

**Methods:**

The study was conducted according to the methodological recommendations of the Preferred Reporting Items for Systematic Reviews and Meta-Analyses (PRISMA) checklist. A systematic literature search was conducted on three search engines, namely PubMed, Scopus and CAB Direct, to identify all publications reporting natural infection of *T. vivax* across the world. All the three search engines were screened using the search term *Trypanosoma vivax* without time and language restrictions. Publications on *T. vivax* that met our inclusion criteria were considered for systematic review and meta-analysis.

**Result:**

The study provides a global database of *T. vivax*, consisting of 899 records from 245 peer-reviewed articles in 41 countries. A total of 232, 6277 tests were performed on 97 different mammalian hosts, including a wide range of wild animals. Natural infections of *T. vivax* were recorded in 39 different African and Latin American countries and 47 mammalian host species. All the 245 articles were included into the qualitative analysis, while information from 186 cross-sectional studies was used in the quantitative analysis mainly to estimate the pooled prevalence. Pooled prevalence estimates of *T. vivax* in domestic buffalo, cattle, dog, dromedary camel, equine, pig, small ruminant and wild animals were 30.6%, 6.4%, 2.6%, 8.4%, 3.7%, 5.5%, 3.8% and 12.9%, respectively. Stratified according to the diagnostic method, the highest pooled prevalences were found with serological techniques in domesticated buffalo (57.6%) followed by equine (50.0%) and wild animals (49.3%).

**Conclusion:**

The study provides a comprehensive dataset on the geographical distribution and host range of *T. vivax* and demonstrates the potential of this parasite to invade other countries out of Africa and Latin America. 
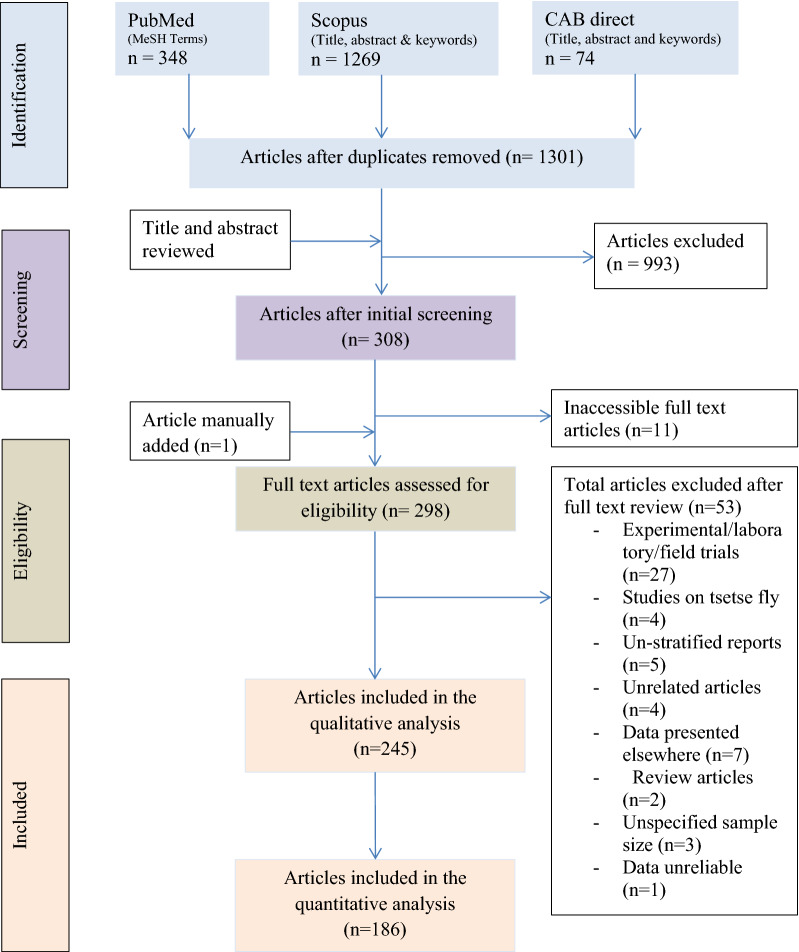

## Introduction

Trypanosomes are protozoan parasites belonging to the family of *Trypanosomatidae* and the genus *Trypanosoma (T.)*. The genus *Trypanosoma* comprises many species such as *T. brucei, T. congolense, T. equiperdum, T. evansi, T. simiae, T. suis* and *T. vivax*, which cause diseases called trypanosomoses in different mammalian hosts including humans [1]. Trypanosomoses are widely distributed in Africa, Latin America and Asia [2, 3].

*Trypanosoma vivax* is one of the most important *Trypanosoma* species known to infect both domestic and wild animals [4, 5]. *Trypanosoma vivax* is reported from cattle, dromedary camel, [6], goat, sheep, pig, dog [7], horse, donkey [8], both domesticated and wild buffalo, warthog, hippopotamus, reedbuck, waterbuck [9], antelope [10], giraffe [11], rhinoceros [12], rodents, pangolins, primates, reptiles and different wild ungulates and carnivores [13]. In Sub-Saharan Africa, *T. vivax* is mainly transmitted by tsetse flies (Diptera: *Glossinidae*) in which the parasite can multiply and remain infective throughout the insect’s life [14]. The parasite has the ability to be transmitted mechanically by hematophagous flies such as *Tabanus* spp., *Stomoxys calcitrans* and *Haematobia irritans,* which are responsible for the spread of *T. vivax* in tsetse-free areas of Africa and in Latin America [4, 15–18]

*Trypanosoma vivax* infection can be suspected by clinical and/or serological evidence and can be confirmed by parasitological or molecular methods [19]. *Trypanosoma vivax* prevalence shows considerable variation with geography, abundance of tsetse or blood-sucking flies, and host species. In tsetse-infested areas of tropical Africa, the *T. vivax* prevalence is typically reported between 5–15% and often accounts for up to half of the total trypanosome prevalence. Outside of the tsetse belt, *T. vivax* prevalence is lower, between 2–10%, and it is related to local and seasonal variation in biting fly abundance [20].

Trypanosomosis caused by *T. vivax* is an important cause of economic losses related to morbidity, mortality, reproductive issues and decreased production [4]. For example, economic losses associated with bovine trypanosomosis have been estimated to be around US$5 billion a year in Africa, and the continent spends at least $30 million every year to control bovine trypanosomosis in terms of curative and prophylactic treatments [21]. Estimates outside Africa indicate that > 11 million head of cattle with a value of > US$ 3 billion are at risk from *T. vivax* infection in the Brazilian Pantanal and Bolivian lowlands, with potential losses in excess of US$ 160 million [16].

Many studies have been conducted on *T. vivax* over the past 100 years. Studies before the 1950s focused more on the morphology and taxonomy [22, 23], pathogenicity [24] and treatment [25, 26]. However, since the 1950s, a considerable number of epidemiological studies have been conducted. Notwithstanding the excellent review on livestock trypanosomoses and their vectors in Latin America [18] and a recent general review on *T. vivax* [20], a systematic literature review on the global distribution, prevalence and host range of *T. vivax* is lacking. Moreover, no information on the global distribution of *T. vivax* is available at the World Animal Health Information System of the World Health Organization (https://www.oie.int/wahis_2/public/wahid.php/Diseaseinformation/Diseasedistributionmap).

Thus, this study was conducted to provide the global distribution of *T. vivax* and to estimate the pooled prevalence of trypanosomosis caused by *T. vivax* in naturally infected domestic and wild animals.

## Methods

The systematic review and meta-analysis were conducted according to the Preferred Reporting Items for Systematic Reviews and Meta-Analyses (PRISMA) checklist [27]. Screening and data extraction were performed by two authors (SL and EF) independently. All disagreements were discussed and resolved by consensus. A third author (PB) was also involved in the search for full-text papers to ensure that all relevant publications were included.

### Literature search

On 30 August 2019, a systematic literature search was conducted on three databases to identify all publications reporting natural infection of *T. vivax* across the world. PubMed, Scopus and CAB Direct were screened using the search term *Trypanosoma vivax* without time and language restrictions. All references found were imported into Mendeley Desktop reference manager software.

### Inclusion and exclusion criteria

To be considered, articles were required to meet the following inclusion criteria: (i) should be observational studies such as cross sectional, longitudinal, case report or outbreak investigation, published in indexed journals, reporting any natural infection of *T. vivax* using any diagnostic test or tests available; (ii) the study design, sample size, sample type, diagnostic methods and number of *T. vivax*-infected animals or prevalence, including 0%; (iii) species of animals with *T. vivax* infections must be provided. Experimental studies; publications which fail to describe diagnostic tools, study design and/or sample sources; and reports solely based on clinical signs were removed despite reporting the prevalence of the disease. In addition, studies reporting *T. vivax* from multiple species without stratifying the report at species level were removed.

### Data extraction

All relevant information such as author names, year of publication, study period, country, region, province, district, latitude, longitude (if provided or if they can be retrieved), host species, number of samples analyzed, type of samples collected, diagnostic method used, number of positives and prevalence or percentage were extracted to a pre-prepared Microsoft Excel spreadsheet (Microsoft Corp., Redmond, WA, USA). When publications only reported the number of animals tested and the prevalence, the numbers of positives were calculated. When publications only reported the number of animals tested and the number of positives, prevalence values were calculated. Publications in other languages than English were translated using Google Translate.

### Data analysis

Owing to heterogeneity within and between studies, random-effects meta-analysis was used to estimate the pooled prevalence and its 95% confidence interval (CI) in different hosts [28]. The estimation was carried out after categorization of the results according to the diagnostic tests used and the host species tested. Accordingly, diagnostic tests were categorized into three categories: (i) parasitological methods, including wet blood smear, stained blood smear and microhematocrit concentration; (ii) serological methods, including enzyme-linked immunosorbent assay (ELISA) both antigen and antibody based, indirect fluorescence antibody test (IFAT) and antigen detection LATEX agglutination; (iii) molecular methods, including reverse line blot hybridization assay, real-time and conventional polymerase chain reaction (PCR). Species-wise, sheep and goat were categorized into “small ruminants,” horse, donkey and mule into “equine” and all studied wild animals including Cape buffalo into “wild animals.” For cattle, domestic buffalo, dromedary camel, pig and dog, pooled prevalence was estimated without categorization.

Heterogeneity between studies was evaluated through the Cochran’s Q test (reported as *p* value), and the inverse variance index (*I*^2^). *I*^2^ describes the percentage of observed total variation between studies due to heterogeneity rather than to random error (intra-study variation). *I*^2^ values < 25% correspond with low heterogeneity, up to 50% with moderate and up to 75% with high heterogeneity [29]. Sub-group analysis using the variable test method was performed to determine the potential sources of heterogeneity among studies. The across-study bias was examined by a funnel plot and Egger’s regression asymmetry test. A funnel plot was used to visually examine the presence of publication bias, and Egger’s regression asymmetry test was used to test whether the bias was statistically significant or not [30]. The unbiased estimates were calculated using the Duval and Tweedie non-parametric ‘fill and trim’ linear random method [31].

The meta-analysis was done using *‘meta’* package of R statistical software version 3.6.2 (R Foundation for Statistical Computing). The map representing the global distribution of *T. vivax* was created, using Quantum GIS software version 3.4.5 (Open Source Geospatial Foundation, Boston, MA, USA).

## Results

### Literature search selection and data extraction

**Fig. 1:**
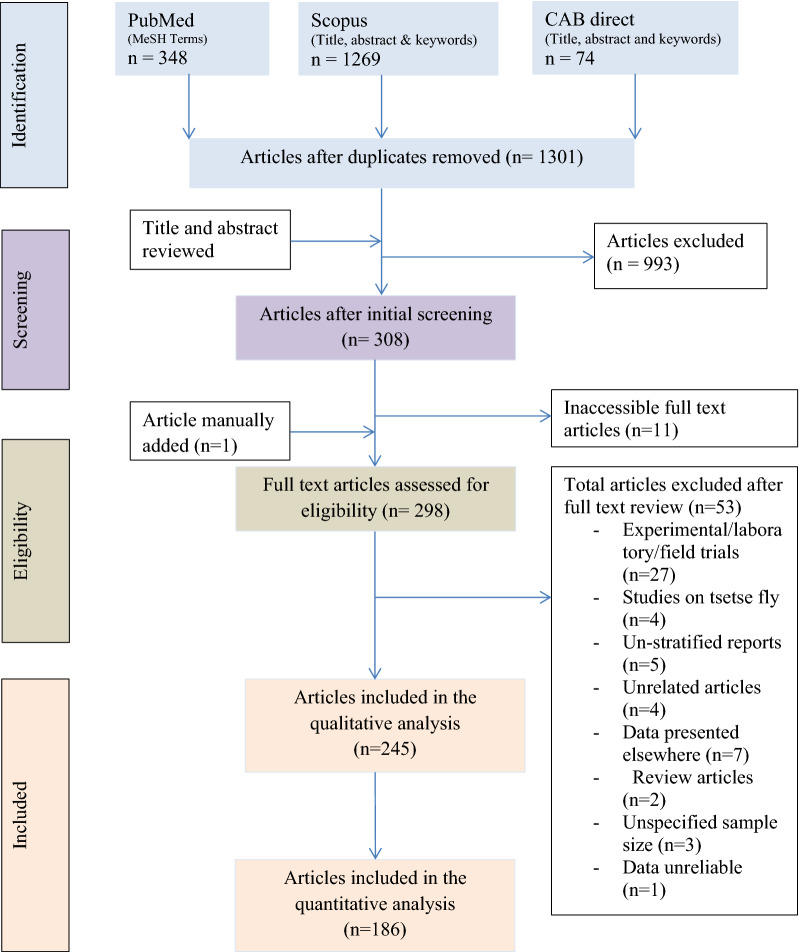
Flow chart representing the selection of studies for inclusion in the systematic review and meta-analysis of global distribution, host range and prevalence of *Trypanosoma vivax*

A total of 1691 publications were retrieved, 348 from PubMed, 1269 from Scopus and 74 from CAB Direct (Fig. 1). After removal of 390 duplicates, the remaining 1301 articles were screened based on their titles and abstracts. Reviews and articles reporting on laboratory and field experiments (*n* = 993) were excluded of further analysis. Articles without an abstract or without sufficient information to make a decision were left for full text review. Of the remaining 308 articles, 11 of the full text files remained inaccessible [32–42]. Finally, one additional article, missed by the systematic literature search, was included manually. Full-text papers of 298 articles were retrieved online or via the library of the Institute of Tropical Medicine Antwerp and eligibility assessed according to the pre-established inclusion/exclusion criteria. Further 53 articles were excluded leaving 245 articles fulfilling all inclusion criteria for the qualitative analysis [4–10, 12–14, 43–277]. Among these 245 articles published between 1958 and 2019, 10 are case reports, 186 report on a cross-sectional study, 35 on a longitudinal study and 14 on an outbreak investigation. All relevant data from these articles were recorded, according to diagnostic method and host species, in a Microsoft Excel file, thus containing 899 records used in the meta-analysis (Additional file 1).

Of these 245 articles, 187 are conducted in 27 African countries, with Ethiopia taking the lead with 43 articles, followed by Nigeria with 29, Uganda with 21 and Kenya with 15 articles. In Latin America, 57 studies were conducted of which 32 were from Brazil, 9 from Venezuela and 6 from Colombia.

### Geographic distribution

**Fig. 2:**
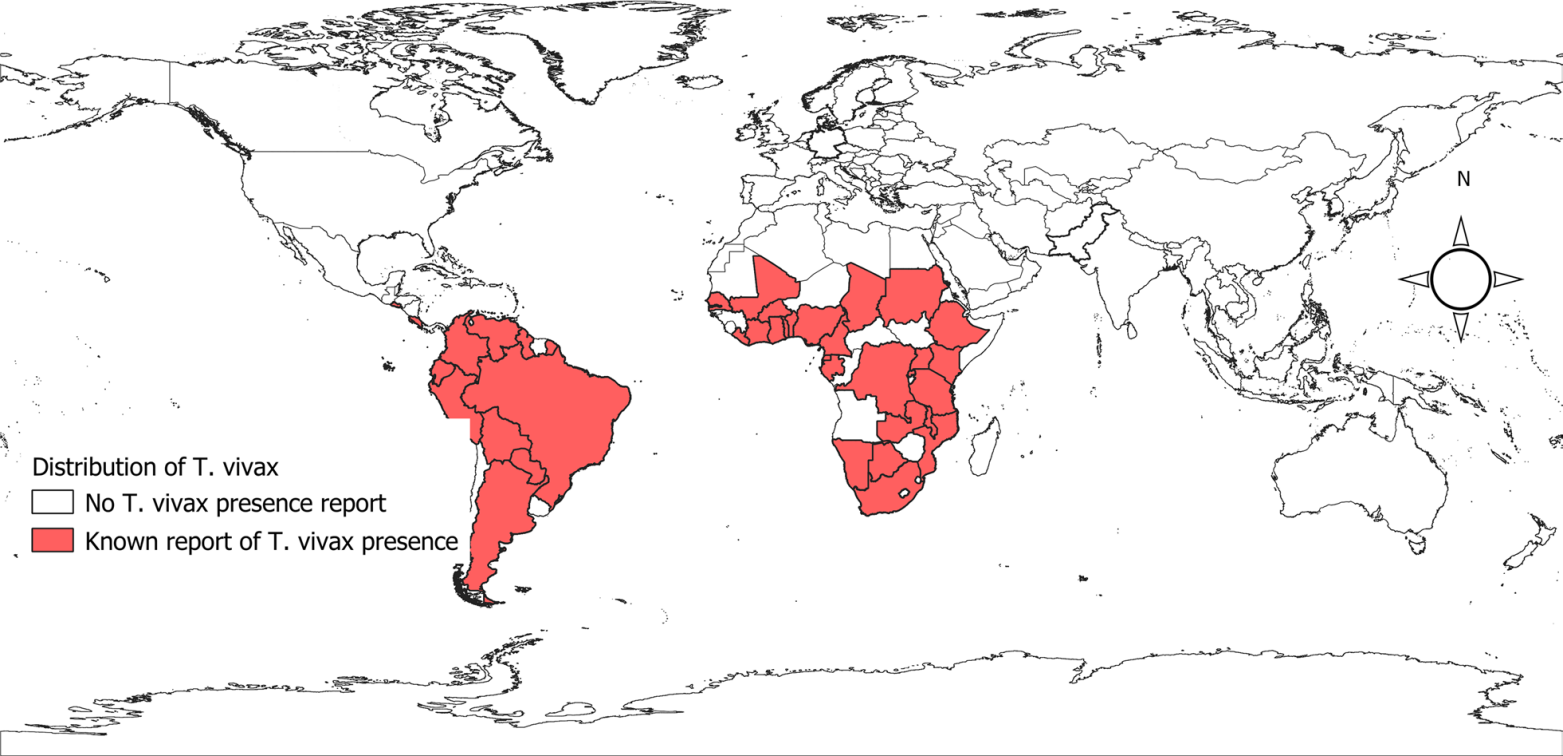
The global distribution of *T. vivax* based on this systematic review including publications between 1958 and 2019

All the studies conducted in the 27 African countries reported the presence of *T. vivax* in at least one host species; natural *T. vivax* infections were found in 12 of the 13 studied Latin American countries (Fig. 2 and Table 1). In Martinique, Alonso and co-workers did not find clinical or serological evidence of *T. vivax* in cattle on this island [50]. One article mentions a cross-sectional study on 300 equines in Pakistan, but all animals were negative in molecular tests for *T. vivax* [231]. We could not find any other reports on the presence of *T. vivax* in Asia, Antarctica, Australia, Europe and North America.

**Table 1:** Countries with reported *T. vivax* infection in diverse host species tested with diverse methods

Country	Host species studied	Test method	Number of tests (total = 232,627)	Number of positives (total =24,420)	References
Argentina	Cattle	Reverse line blot	186	16	[217]
Benin	Buffoon kob, cattle, hartebeest, roan antelope, warthog, waterbuck,	Thin and thick blood smears	312	205	[10, 99]
Bolivia	Cattle	Thin blood smear, Giemsa stained blood smear, PCR	1520	311	[123, 177, 243]
Botswana	Cape buffalo, cattle, donkey, goat, greater kudu, impala, lechwe, reedbuck, sable antelope, tsessebe	MHCT/Woo test, Giemsa stained thick and thin blood smear, IFAT	3040	399	[100, 239]
Brazil	Cattle, donkey, goat, horse, pampas deer, sheep, domestic buffalo	MHCT/Woo test, PCR, IFAT, thin and thick blood smear, buffy coat smear, Ab-ELISA, PCR	11468	4079	[4, 51, 58–62, 71, 72, 81, 83–85, 91, 94, 112, 116, 125–127, 154, 156, 212, 219, 220, 226, 228, 242, 244, 250, 274, 277]
Burkina Faso	Cattle	Buffy coat smear, Ag-ELISA, Ab-ELISA, PCR	11095	1095	[65, 90, 151, 216, 241, 251–253]
Cameroon	African civet, black legged mangoose, black striped duiker, blue duiker, bosman potto, brush tailed porcupine, cattle, cloaked mangabey, crested mangabey, crocodile, dark mangoose, de Brazza’s monkey, dog, dwarf guenon, giant forest squirrel, giant rat, goat, golden cat, golden potto, greater cane rat, greater white-nosed monkey, guereza white colobus, long-tailed pangolin, mandrill, mona monkey, monitor lizard, moustached monkey, ogilby’s duiker, Peter’s duiker,pig, red-legged sun squirrel, royal antelope, sheep, sitatunga, small-spotted genet, tree dassie, tree pangolin, two-spotted palm civet, water chevrotain, white-eyelid mangabey, yellow-backed duiker	Buffy coat smear, PCR	4176	406	[7, 13, 14, 170, 202, 245] [7, 13, 14, 170, 202, 245]
Chad	Cattle	Buffy coat smear, Ab-ELISA	1866	435	[93]
Colombia	Cattle, goat, sheep	Blood smear, PCR, IFAT	6712	1699	[135, 136, 215, 221, 232, 274]
Costa Rica	Cattle	Blood smear, IFAT	642	53	[210, 274]
Côte d’Ivoire	Cattle, goat, pig, sheep	MHCT/Woo test, PCR	2185	195	[45, 148, 197]
Democratic Republic of the Congo	Cattle, dog, goat, pig, sheep	MHCT/Woo test, ELISA	685	41	[167, 168]
Ecuador	Cattle	IFAT	310	70	[274]
El Salvador	Cattle	IFAT	100	15	[274]
Equatorial Guinea	Goat, sheep	PCR	559	10	[79]
Ethiopia	Cattle, donkey, dromedary camel, goat, horse, mule, sheep	Giemsa stained blood smear, blood smear, buffy coat smear, MHCT/Woo test, thin and thick blood smear, Ab-ELISA, PCR	55196	2600	[6, 43, 44, 48, 64, 69, 70, 74–76, 86, 87, 92, 98, 101, 103, 107, 113–115, 122, 142, 144, 145, 152, 178–182, 184, 187, 191, 229, 230, 240, 248, 257, 258, 262, 264–266]
French Guiana	Cattle	Ag-ELISA	3000	870	[95]
Gabon	Cattle	Buffy coat smear, Ag-ELISA, PCR	442	26	[80, 157, 268]
Gambia	Cattle, donkey, goat, horse, sheep	Buffy coat smear, Giemsa stained blood smear, Ab-ELISA, PCR	5745	1329	[8, 96, 102, 173, 213, 218]
Ghana	Cattle, goat, pig, sheep	Buffy coat smear, PCR, Ag-latex agglutination test	1786	231	[46, 117, 143, 198]
Guyana	Goat, sheep	MHCT/Woo test, IFAT	467	15	[55, 272]
Kenya	Black rhinoceros, cattle, dromedary camel, goat, horse, pig, sheep	Thin and thick blood smear, Giemsa stained blood smear, buffy coat smear, MHCT/Woo test, Ag-ELISA, PCR	5156	845	[66, 146, 172, 174, 183, 185, 196, 200, 201, 208, 209, 227, 261, 267, 275]
Liberia	Cattle	Giemsa stained blood smear, IFAT, Ab-ELISA	700	327	[155, 176]
Malawi	Cattle	Giemsa stained blood smear	9309	9	[271]
Mali	Cattle	Buffy coat smear	796	34	[192]
Martinique	Cattle	IFAT	227	0	[50]
Mozambique	Cattle	Blood smear	16895	1245	[254]
Namibia	Cattle	Giemsa stained thick and thin blood smear, MHCT	1481	15	[270]
Nigeria	Cattle, dog, goat, horse, sheep	Blood smear, Giemsa stained blood smear, MHCT/buffy coat smear, MHCT/Woo test, Ag-ELISA, PCR	20080	2926	[52, 53, 63, 88, 89, 104–106, 108–110, 134, 137–140, 153, 166, 206, 207, 211, 214, 234–237, 259, 269, 276]
Pakistan	Donkey, horse, mule	PCR	300	0	[231]
Paraguay	Cattle	IFAT	15	6	[274]
Peru	Cattle	Blood smear, MHCT/Woo test, Giemsa stained blood smear, IFAT, PCR	985	119	[171, 177, 222, 274]
Rwanda	Cattle	Blood smear	3630	36	[12]
Senegal	Cattle, dog, donkey, goat, horse, sheep	Buffy coat smear, blood smears, Ab-ELISA, PCR	4890	365	[111, 128, 129, 225, 238]
South Africa	Cattle	PCR	143	30	[169]
Sudan	Cattle, donkey, dromedary camel, horse	Blood smear, Buffy coat smear, PCR	4426	366	[132, 186, 223, 233]
Tanzania	African civet, bohor reedbuck, cattle, Coke’s hartebeest, giraffe, Grant’s gazelle, hunting dog, impala, Kirk’s dikdik, klipspringer, Lichtenstein’s hartebeest, oribi, oryx, ostrich, pig, roan antelope, southern reedbuck, steinbuck, Thomson’s gazelle, tsessebe, warthog, wildebeest, zebra	Blood smear, thin and thick blood smear, Giemsa stained blood smear, buffy coat smear, PCR, PCR-LAMP	9974	431	[5, 56, 78, 130, 131, 133, 141, 147, 149, 188, 194, 203, 247, 256]
Togo	Cattle	PCR-RFLP	354	27	[263]
Uganda	Cattle, dog, donkey, goat, pig, sheep	Giemsa stained blood smear, MHCT/Woo test, thick and thin blood smear, buffy coat smear, Ab-ELISA, PCR	28510	1932	[47, 49, 54, 57, 67, 68, 77, 82, 158–165, 189, 190, 195, 205, 273]
Venezuela	Cattle, horse, sheep, domestic buffalo	MHCT/Woo test, stained blood smear, IFAT, Ab-ELISA, PCR	6328	1373	[73, 118–121, 124, 224, 255, 260]
Zambia	African civet, baboon, bat, black rhinoceros, bushbuck, cane rat, Cape buffalo, cattle, crocodile, eland, elephant, genet, giraffe, goat, greater kudu, grey duiker, grysbok, hare, hartebeest, hippopotamus, hunting dog, hyena, impala, jackal, leopard, lion, mongoose, pig, porcupine, puku, reedbuck, roan antelope, serval, vervet monkey, warthog, waterbuck, wild cat, wildebeest, zebra	PCR, buffy coat smear	6936	234	[9, 97, 150, 175, 193, 199, 204, 246]

*Ab-Elisa* antibody enzyme-linked immunosorbent assay, *Ag-Elisa* antigen enzyme-linked immunosorbent assay, *Mhct* micro-hematocrit centrifugation technique, *Ifat* immunofluorescence antibody test, *Pcr* polymerase chain reaction, *Pcr-Lamp* polymerase chain reaction-loop mediated isothermal amplification, *Pcr-Rflp* polymerase chain reaction-restriction fragment length polymorphism

### Host range

A total of 232,627 tests were performed, and 24,420 of them were positive for natural infection of *T. vivax*. *Trypanosoma vivax* was reported from nine domestic animal species: cattle, domestic buffalo, dog, donkey, dromedary camel, goat, horse, pig and sheep. Among them, cattle were the most studied species with 198,593 tests performed on cattle in 36 countries and two territories (192 publications) and 20,964 were positive for *T. vivax*. Next to cattle, goat, sheep, pig and donkey were the most frequently studied species. The protozoal parasite was also reported from wild animals including diverse species of antelopes, Cape buffalo, hippopotamus, black rhinoceros, pangolin and warthog. *Trypanosoma vivax* was reported from 39 wild fauna species, including many antelope species and Cape buffalo (Tables 2, 3).

**Table 2:** Domestic animal species tested for infection with *T*. *vivax*

Species	List of countries	Number of tests	Positive animals	References
Cattle	Argentina, Benin, Bolivia, Botswana, Brazil, Burkina Faso, Cameroon, Chad, Colombia, Costa Rica, Côte d’Ivoire, Democratic Republic of the Congo, Ecuador, El Salvador, Ethiopia, French Guiana, Gabon, Gambia, Ghana, Kenya, Liberia, Malawi, Mali, Martinique^a^, Mozambique, Namibia, Nigeria, Paraguay, Peru, Rwanda, Senegal, South Africa, Sudan, Tanzania, Togo, Uganda, Venezuela, Zambia	198593	20964	[4–6, 12, 14, 40, 44–54, 57–59, 61, 62, 64–66, 68–71, 73–78, 80–83, 85–88, 90–95, 99, 101–103, 105, 106, 108, 109, 111, 113, 114, 116, 118, 122, 123, 125–127, 129–132, 134–136, 138, 139, 141–143, 145, 147–153, 155–159, 161–167, 169–171, 173–177, 180, 182, 184, 185, 187–200, 202, 203, 205–212, 214–217, 219, 220, 222–227, 229, 230, 232, 235, 236, 238–243, 246–259, 261–271, 273, 274, 276–282]
Dromedary camel	Ethiopia, Kenya, Sudan	1611	133	[6, 115, 172, 186]
Dog	Cameroon, Democratic Republic the Congo, Nigeria, Senegal, Uganda	574	1	[7, 137, 158, 168, 214, 225]
Donkey	Botswana, Brazil, Ethiopia, Pakistan^a^, Sudan, Uganda	2713	152	[6, 8, 43, 96, 107, 178, 189, 225, 228, 231, 233, 239, 248]
Goat	Botswana, Brazil, Cameroon, Colombia, Côte d’Ivoire, Democratic Republic of the Congo, Equatorial Guinea, Ethiopia, Gambia, Ghana, Guyana, Kenya, Nigeria, Senegal, Uganda, Zambia	9715	526	[6, 7, 55, 57, 60, 63, 68, 79, 98, 112, 128, 137, 140, 143, 144, 150, 158, 168, 179, 193, 197, 201, 204, 211, 213, 218, 221, 225, 234, 237, 239, 246, 248]
Horse	Brazil, Ethiopia, Gambia, Kenya, Nigeria, Pakistan^a^, Senegal, Sudan, Venezuela	3305	857	[8, 84, 96, 104, 118, 146, 181, 214, 225, 231, 233]
Mule	Ethiopia, Pakistan^a^	353	0	[43, 181, 231, 248]
Pig	Cameroon, Côte d’Ivoire, Democratic Republic the Congo, Ghana, Kenya, Tanzania, Uganda, Zambia	2650	233	[7, 57, 67, 68, 133, 158, 168, 197, 198, 201, 245, 246]
Sheep	Brazil, Cameroon, Colombia, Côte d’Ivoire, Democratic Republic of the Congo, Equatorial Guinea, Ethiopia, Gambia, Ghana, Guyana, Kenya, Nigeria, Senegal, Uganda, Venezuela	6447	455	[6, 7, 55, 57, 60, 79, 98, 116, 118, 120, 128, 134, 137, 140, 143, 144, 168, 197, 201, 213, 221, 225, 234, 248, 272]
Small ruminants	Kenya, Nigeria	988	69	[89, 110, 227]
Domestic buffalo	Brazil, Venezuela	2144	509	[116, 118, 119, 121, 260]

^a^*T. vivax* was not observed in Martinique and Pakistan

**Table 3:** Wild animal species tested positive for *T. vivax* infection

Host species	Scientific name	Country	Number of tests	Positive tests	Positivity rate	References
Black rhinoceros	*Diceros bicornis*	Kenya	1	1	100	[283]
Black striped duiker	*Cephalophus dorsalis*	Cameroon	37	3	8.1	[13]
Blue duiker	*Cephalophus monticola*	Cameroon	290	24	8.3	[13]
Bosman potto	*Perodicticus potto*	Cameroon	8	3	37.5	[13]
Brush tailed porcupine	*Atherurus africanus*	Cameroon	106	7	6.6	[13]
Buffoon kob	*Kobus kob*	Benin	50	1	2	[10]
Bushbuck	*Tragolaphus scriptus*	Zambia	51	4	7.8	[97]
Cape buffalo	*Syncerus caffer*	Botswana, Zambia	1105	285	25.8	[9, 97, 100]
Cloaked mangabey	*Cercocebus albigena*	Cameroon	12	2	16.7	[13]
Crocodile	*Crocodylus niloticus*	Cameroon	3	1	33.3	[13]
De Brazza’s Monkey	*Cercopithecus neglectus*	Cameroon	1	1	100	[13]
Dwarf guenon	*Miopithecus tlapoin*	Cameroon	55	5	9.1	[13]
Eland	*Taurotragus oryx*	Zambia	3	1	33.3	[97]
Giant rat	*Cricetomys gambianus*	Cameroon	135	4	2.9	[13]
Greater kudu	*Tragelaphus strepsiceros*	Botswana, Zambia	36	26	72.2	[97, 100]
Greater white-nosed monkey	*Cercopithecus nictitans*	Cameroon	155	22	14.2	[13]
Grey duiker	*Sylvicapra grimmia*	Zambia	7	1	14.3	[97]
Guereza white colobus	*Colobus guereza*	Cameroon	14	2	14.3	[13]
Hartebeest	*Alcelaphus bubalis*	Benin	20	1	5	[10]
Hippopotamus	*Hippopotamus amphibius*	Zambia	29	1	3.4	[9]
Impala	*Aepyceros melampus*	Botswana	23	14	60.9	[100]
Lechwe	*Kobus leche*	Botswana	110	39	35.5	[100]
Long tailed pangolin	*Manis tetradactyla*	Cameroon	34	2	5,9	[13]
Mona monkey	*Cercopithecus mona*	Cameroon	46	8	17,4	[13]
Monitor lizard	*Varanus ornatus*	Cameroon	8	1	12,5	[13]
Moustached monkey	*Cercopithecus cephus*	Cameroon	101	11	10.9	[13]
Oryx	*Oryx beisa*	Tanzania	1	1	100	[56]
Puku	*Kobus vardonii*	Zambia	24	1	4.2	[97]
Reedbuck	*Redunca sp.*	Botswana, Zambia	3	3	100	[9, 100]
Sable antelope	*Hippotragus niger*	Botswana	22	7	31.8	[100]
Sitatunga	*Tragelaphus spekei*	Cameroon	5	1	20	[13]
Small-spotted genet	*Genetta servalina*	Cameroon	8	1	12.5	[13]
Southern reedbuck	*Redunca arundinum*	Tanzania	4	1	25	[56]
Tree pangolin	*Manis tricuspis*	Cameroon	20	5	25	[13]
Tsessebe	*Damaliscus lunatus*	Botswana	15	6	40	[100]
Two-spotted palm civet	*Nandinia binotata*	Cameroon	32	3	9.4	[13]
Warthog	*Phacochoerus aethiopicus*	Zambia	56	1	1.8	[9]
Waterbuck	*Kobus ellipsiprymnus*	Zambia	30	19	63.3	[9, 97]
White-eyelid mangabey	*Cercocebus torquatus*	Cameroon	5	2	40	[13]

### Pooled prevalence estimates according to host species and type of diagnostic test

Pooled prevalence estimates by test methods for different hosts are presented in Table 4, and forest plots of the meta-analysis and the subgroup analyses can be found in Additional files 2 and 3. Substantial heterogeneity was observed in the pooled estimate except for dog, which remained significant (*P* < 0.05) even after sub-group analysis.

**Table 4:** Sub-group meta-analysis for different species using different diagnostic methods

Host species	Diagnostic method	Number of publications	Number of tests	Number of positives	Pooled prevalence in %	95% CI
Domestic buffalo	Parasitological	1	316	36	11.4	8.3–15.4
	Molecular	2	609	127	20.9	17.4–25.0
	Serological	2	556	301	57.6	22.5–86.4
Camel	Molecular	4	1611	133	8.4	3.4–19.3
Cattle	Parasitological	92	102910	5414	4.6	4.0–5.3
	Molecular	51	31549	3140	7.4	6.2–8.7
	Serological	23	16469	4495	34.6	28.0–41.9
Dog	Parasitological	3	257	0	3.4	1.1–9.6
	Molecular	2	189	1	1.2	0.2–8.5
Domestic buffalo	Parasitological	1	316	36	11.4	8.3–15.4
	Molecular	2	609	127	20.9	17.4–25.0
	Serological	2	556	301	57.6	22.5–86.4
Equine	Parasitological	8	2471	20	1.5	0.9–2.6
	Molecular	7	1425	251	5.6	2.7–11.3
	Serological	1	6	3	50	16.8–83.2
Pig	Parasitological	4	799	3	1.1	0.4–2.9
	Molecular	10	1851	230	9	4.9–15.9
Small ruminant	Parasitological	19	8990	220	2.3	1.5–3.6
	Molecular	15	4045	327	5	2.7–9.3
	Serological	3	408	43	13.8	6.1–28.4
Wild animal	Parasitological	3	1093	75	11.8	7.1–16.9
	Molecular	3	1618	121	10.7	8.6–13.3
	Serological	1	748	318	49.3	37.5–61.2

A total of 145 cross-sectional studies from 32 countries were included in estimation of natural infection of *T. vivax* in cattle. The random effect model indicates the pooled prevalence to be 6.4% (5.7–7.2, 95% CI). For small ruminants, pooled prevalence of *T. vivax* was estimated from 33 studies in 16 countries and found to be 3.8% (2.5–5.6, 95% CI). A total of 15 studies from 10 different countries were used to estimate the pooled prevalence of *T. vivax* in equines. The random effect model estimates the pooled prevalence to be 3.7% (2.0–6.8, 95% CI). Pooled prevalence of *T. vivax* in camels was estimated from four studies in three different countries. The model estimates a pooled prevalence of 8.4% (3.4–19.3, 95% CI). A total of 12 studies from 8 different countries were included in the estimation of pooled prevalence in pigs, which was found to be 5.5% (3.0–10.1, 95% CI). Five studies from five countries were used in the estimation pooled prevalence of *T. vivax* in dogs. The pooled prevalence was estimated to be 2.6% (1.0–6.3% 95% CI). Three studies reported natural infection of *T. vivax* in domestic buffaloes from Venezuela, and the random effect model estimates a pooled prevalence of 30.6% (14.2–54.1, 95% CI). For wild animals, a pooled prevalence of 12.9% (9.9–16.6, 95% CI) was estimated from six studies in five countries. Subgroup pooled prevalences estimated according to the type of diagnostic test, as represented in Table 4, were lowest with parasitological techniques (from 1.1% in pigs to 13.2% in wild animals) and highest with serological techniques (from 13.8% in small ruminants to 57.6% in domestic buffalo).

### Publication bias

**Fig. 3:**
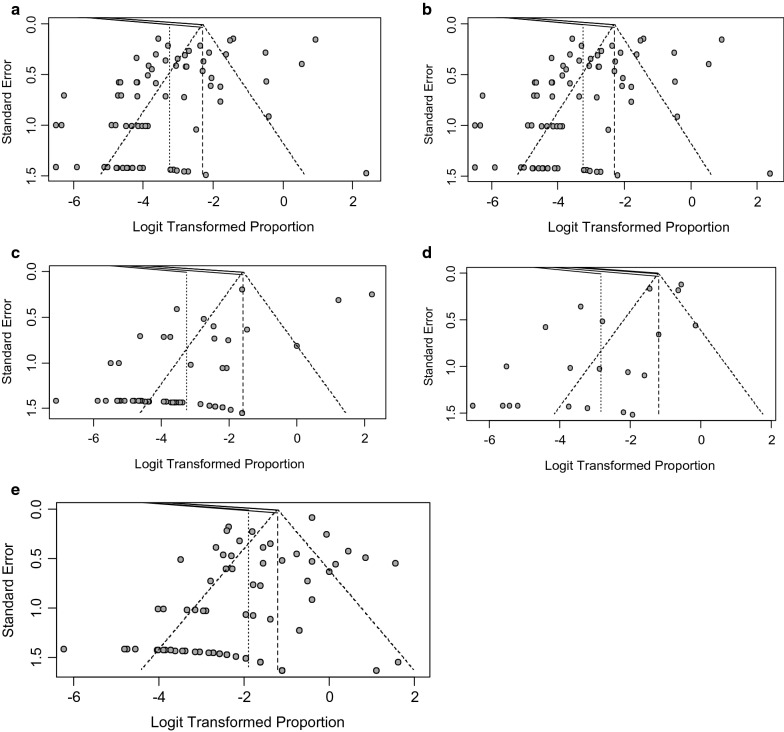
Publication bias evidenced by funnel plots for cattle (**a**), small ruminants (**b**), equines (**c**), pigs (**d**) and wild animals (**e**)

The presence of publication bias was analyzed only in five species since there were not enough publications to discuss its possible influence in camel, domestic buffalo and dogs. Possible publication bias was demonstrated by visualization of asymmetry in funnel plots for cattle (Fig. 3a), small ruminants (Fig. 3b), equines (Fig. 3c), pigs (Fig. 3d) and wild animals (Fig. 3e). It was further confirmed by ‘*metabias*’ test (Egger’s test) with *p*-value < 0.05. The '*trimfill*' method imputed 170, 43, 30, 27 and 11 studies to obtain symmetry in funnel plots in cattle, wild animals, equines, small ruminants and pigs, respectively. The new estimated prevalence equals to 14.8% for cattle, 26.8% for wild animals, 21.6% for equines, 9.5% for small ruminants and 24.5% for pigs.

## Discussion

This study presents the first systematic review of published literature since the 1950s describing global distribution, host range and prevalence of trypanosomosis caused by *T. vivax*. Not surprisingly, most publications report on *T. vivax* infections in domestic mammalian species, in particular in cattle and small ruminants, while few publications describe natural infections in wildlife.

Looking at the *T. vivax* distribution map (Fig. 2), there is an evident data gap for some sub-Saharan African countries where tsetse flies are present and therefore *T. vivax* may be endemic. Although our formal search strategy could not retrieve any publication on these "missing" countries, conventional Google search confirms the presence of *T. vivax* in South Sudan and Zimbabwe [284, 285], and Genevieve et al. [286] reported on the presence of potential vectors in the Central African Republic. Since Angola, the Central African Republic and the Republic of Congo are endemic for human African trypanosomosis, the presence of *T. vivax* in these countries is likely [287]. Due to its adaptation to mechanical transmission, *T. vivax* is also present outside the tsetse belt in Africa, e.g. in Ethiopia and Sudan [114, 288]. As a consequence, the trypanosomosis control efforts with focus on tsetse eradication might have little effect on *T. vivax*. Also, economic impact assessments that are solely based on tsetse distribution alone could seriously underestimate the problem of trypanosomosis because of *T. vivax*.

Out of Africa, *T. vivax* is present in Latin America but not in North America, Australia, Asia and the Pacific regions. *Trypanosoma vivax* is believed to be introduced into Latin America in cattle and horses imported from Africa, possibly in the sixteenth century, and spread to different Latin American countries including Brazil, Colombia, French Guiana, Guadeloupe, Guyana, Martinique, Panama, Suriname and Venezuela [18]. Stephen [289] reviewed the presence of the parasite in Costa Rica, Ecuador, El Salvador, Paraguay and Peru, and according to Gardiner et al. [15], *T. vivax* was present in the Caribbean thus posing a threat to the livestock industries. From our literature search, we can only confirm *T. vivax* to be endemic in 12 Latin American countries of which 7 (Argentina, Bolivia, Brazil, Colombia, Guyana, Peru, Venezuela) are also endemic for *T. evansi* [290]. Although, our literature search provides information on the potential spread of *T. vivax* in Latin America, it is important to note that the distribution could be much wider, for example, *T. vivax* was only detected in Argentina in 2018; this is this due to the lack of previous studies. Apparently, *T. vivax* has never spread into Asia, unlike *T. evansi*, although similar to the latter; it can be mechanically transmitted by bloodsucking flies. Unless there is a particular biological or environmental factor preventing *T. vivax* from invading the Middle East and Asia, as well Northern Africa, North America and Europe, we must remain alert about the risk of importing *T. vivax* into non-endemic countries as happened in Latin America.

This review suggests that *T. vivax* has a very diverse host range, including 9 domestic mammals and almost 40 wild fauna species. Regarding the latter, however, data should be interpreted with caution. Diagnostic tests, whether parasitological, serological or even molecular, have their limitations. For examples, by sequencing of PCR amplicons, Auty and co-workers [11] clearly demonstrate that wildlife may harbor a diversity of trypanosomes, including taxonomically undefined species. Therefore, it is likely that many reports on *T. vivax* infection in wildlife and tsetse in fact deal with other trypanosome species that are not necessarily pathogenic for domestic animals.

The pooled prevalence of trypanosomosis in different hosts varies significantly depending on the detection methods; significantly higher estimates were reported in publications using serological techniques. Higher estimates using a serological technique could be due to the persistence of the antibody over several months after curative treatment and the possibility of low undetectable parasitemia in parasitological techniques [20, 93, 291, 292]. Moti et al. [187] compared the percentage positivity obtained with different diagnostic techniques and showed that relative to the microhematocrit centrifugation technique the percent positivity increased by 50 and 250% when using PCR-RFLP. Also, Garcia et al. [118] reported that for the detection of trypanosomes, PCR-based assays are twice as sensitive as parasitological techniques such as the microhaematocrit centrifugation.

The study has the following limitations. The literature search was almost exclusively based on electronic databases whereby some older literature must have been missed. The data showed a large degree of heterogeneity among studies, which remain significant after sub-group analysis. There is a significant publication bias, which could be due to incomplete or inaccurate information provided in the publications. In addition, studies were conducted between 1956 and 2017, and the result may not accurately reflect the current epidemiological situation and therefore could limit interpretation of the result to some degree. Furthermore, we suspect numerous data gaps mainly because of two reasons. First, due to the lack of a country-level monitoring and reporting system for trypanosomosis, most of the data included in this analysis are from research activities. Second, trypanosomosis diagnosis in most endemic countries relied to a great extent on low-sensitivity parasitological methods, while more sensitive molecular tools are rarely used. In addition, the majority of studies analyzing trypanosome's presence in the field may not have a sampling strategy that allows a robust estimation of prevalence. This is for multiple and understandable reasons—samples can be difficult and expensive to collect, and many studies rely on purposive sampling, or sampling of, for example, animals presented to veterinary clinics. While these kinds of studies provide a rough idea of pathogen presence/absence, they may not provide an accurate estimate of prevalence. Thus, caution should be taken when interpreting the results presented here.

## Conclusion

With this study, we intended to provide comprehensive information on the geographical distribution, host range and prevalence of trypanosomosis caused by *T. vivax* worldwide. The results confirm the wide geographical distribution and a diverse host range of *T. vivax*. The parasite parasitizes almost all domestic mammals and many wild animal species, thus suggesting the potential to get established in other countries with favorable environmental conditions, e.g. in the Middle East, Asia and Australia. The meta-analysis showed a high degree of variability in estimated prevalence values. The variability can be attributed to diagnostic tests used and the species of the animal infected.

## Supplementary Information

**Additional file 1.** Global *Trypanosoma vivax* occurrence records.

**Additional file 2.** Forest plots showing an overview of studies reporting *Trypanosoma vivax* in different host species.

**Additional file 3.** Forest plots showing an overview of studies reporting *Trypanosoma vivax* grouped by test methods in different host species.

## Data Availability

All data analyzed in this paper are provided as supplementary file.
