# Supplementary material for: Global distribution, host range and prevalence of Trypanosoma vivax: a systematic review and meta-analysis
Source: Parasit Vectors. 2021 Jan 25;14:80. doi: 10.1186/s13071-021-04584-x (PMC7830052; doi:10.1186/s13071-021-04584-x)

camel

Study	Events	Total	Weight (fixed)	Weight (random)	IV, Fixed + Random, 95% CI
Birhanu et al 2015	16	343	14.9%	14.2%	0.05 [0.03; 0.07]
Birhanu et al 2015	10	411	9.6%	13.9%	0.02 [0.01; 0.04]
Masiga and Nyang'ao 2001	0	80	0.5%	6.6%	0.00 [0.00; 0.05]
Mossaad et al 2017	10	41	7.4%	13.7%	0.24 [0.12; 0.40]
Mossaad et al 2017	10	41	7.4%	13.7%	0.24 [0.12; 0.40]
Mossaad et al 2017	37	148	27.2%	14.5%	0.25 [0.18; 0.33]
Mossaad et al 2017	49	148	32.1%	14.5%	0.33 [0.26; 0.41]
Regassa et al 2015	1	399	1.0%	9.1%	0.00 [0.00; 0.01]
**Total (fixed effect, 95% CI)**		**1611**	**100.0%**	**--**	**0.17 [0.15; 0.20]**
**Total (random effects, 95% CI)**			**--**	**100.0%**	**0.08 [0.03; 0.19]**

Heterogeneity: $Tau^2 = 1.6066$; $Chi^2 = 135.14$, df = 7 ($P < 0.01$); $I^2 = 95\%$

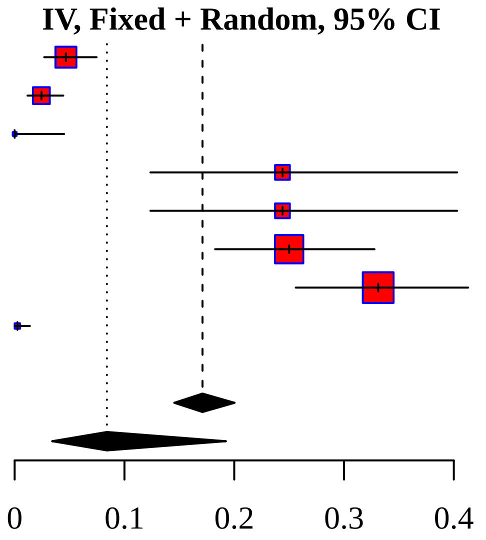

Cattle

Study	Events	Total	Weight (fixed)	Weight (random)	IV, Fixed + Random, 95% CI
Abebe et al 2017	24	1508	0.2%	0.3%	0.02 [0.01; 0.02]
Acapovi−Yao et al 2016	142	1270	1.3%	0.3%	0.11 [0.09; 0.13]
Adam et al 2012	1	50	0.0%	0.2%	0.02 [0.00; 0.11]
Adam et al 2012	1	50	0.0%	0.2%	0.02 [0.00; 0.11]
Adam et al 2012	1	50	0.0%	0.2%	0.02 [0.00; 0.11]
Adam et al 2012	2	50	0.0%	0.2%	0.04 [0.00; 0.14]
Adam et al 2012	2	50	0.0%	0.2%	0.04 [0.00; 0.14]
Adam et al 2012	1	50	0.0%	0.2%	0.02 [0.00; 0.11]
Adam et al 2012	1	50	0.0%	0.2%	0.02 [0.00; 0.11]
Adam et al 2012	1	50	0.0%	0.2%	0.02 [0.00; 0.11]
Adam et al 2012	3	50	0.0%	0.2%	0.06 [0.01; 0.17]
Adam et al 2012	3	50	0.0%	0.2%	0.06 [0.01; 0.17]
Adam et al 2012	1	50	0.0%	0.2%	0.02 [0.00; 0.11]
Adam et al 2012	1	50	0.0%	0.2%	0.02 [0.00; 0.11]
Adam et al 2012	6	50	0.1%	0.2%	0.12 [0.05; 0.24]
Ahmed et al 2013	17	600	0.2%	0.3%	0.03 [0.02; 0.04]
Ahmed et al 2013	34	600	0.3%	0.3%	0.06 [0.04; 0.08]
Ali and Bitew 2011	13	385	0.1%	0.3%	0.03 [0.02; 0.06]
Alingu et al 2014	6	295	0.1%	0.2%	0.02 [0.01; 0.04]
Alonso et al 1992	0	227	0.0%	0.1%	0.00 [0.00; 0.02]
Anene et al 1991a	1	68	0.0%	0.2%	0.01 [0.00; 0.08]
Anene et al 1991a	3	290	0.0%	0.2%	0.01 [0.00; 0.03]
Anene et al 1991b	28	102	0.2%	0.3%	0.27 [0.19; 0.37]
Angwech et al 2015	260	816	1.8%	0.3%	0.32 [0.29; 0.35]
Balyeidhusa et al 2012	1	5	0.0%	0.1%	0.20 [0.01; 0.72]
Balyeidhusa et al 2012	3	83	0.0%	0.2%	0.04 [0.01; 0.10]
Balyeidhusa et al 2012	5	117	0.0%	0.2%	0.04 [0.01; 0.10]
Balyeidhusa et al 2012	8	119	0.1%	0.2%	0.07 [0.03; 0.13]
Bejano et al 2016	7	322	0.1%	0.2%	0.02 [0.01; 0.04]
Bengaly et al 1995	0	50	0.0%	0.1%	0.00 [0.00; 0.07]
Bengaly et al 1995	93	1633	0.9%	0.3%	0.06 [0.05; 0.07]
Bengaly et al 1995	248	1633	2.2%	0.3%	0.15 [0.13; 0.17]
Birhanu et al 2015	13	411	0.1%	0.3%	0.03 [0.02; 0.05]
Biryomumaisho et al 2013	8	500	0.1%	0.3%	0.02 [0.01; 0.03]
Biryomumaisho et al 2013	11	584	0.1%	0.3%	0.02 [0.01; 0.03]
Biryomumaisho et al 2013	19	807	0.2%	0.3%	0.02 [0.01; 0.04]
Bishaw et al 2012	24	384	0.2%	0.3%	0.06 [0.04; 0.09]
Bitew et al 2011	7	100	0.1%	0.2%	0.07 [0.03; 0.14]
Bitew et al 2011	4	100	0.0%	0.2%	0.04 [0.01; 0.10]
Bitew et al 2011	5	100	0.0%	0.2%	0.05 [0.02; 0.11]
Bittar et al 2015	9	44	0.1%	0.2%	0.20 [0.10; 0.35]
Bittar et al 2015	15	44	0.1%	0.3%	0.34 [0.20; 0.50]
Camejo et al 2016	50	85	0.2%	0.3%	0.59 [0.48; 0.69]
Chanie et al 2012	24	384	0.2%	0.3%	0.06 [0.04; 0.09]
Clausen et al 1998	13	181	0.1%	0.3%	0.07 [0.04; 0.12]
Clausen et al 1998	13	486	0.1%	0.3%	0.03 [0.01; 0.05]
Connor and Halliwell 1987	16	1617	0.2%	0.3%	0.01 [0.01; 0.02]
Connor and Halliwell 1987	16	1617	0.2%	0.3%	0.01 [0.01; 0.02]
Costa et al 2013	0	509	0.0%	0.1%	0.00 [0.00; 0.01]
Dagnachew et al 2011	12	300	0.1%	0.3%	0.04 [0.02; 0.07]
Dagnachew et al 2017	3	640	0.0%	0.2%	0.00 [0.00; 0.01]
Dagnachew et al 2017	2	795	0.0%	0.2%	0.00 [0.00; 0.01]
De Araujo Melo et al 2011	3	559	0.0%	0.2%	0.01 [0.00; 0.02]
Delafosse et al 2006	403	933	2.4%	0.3%	0.43 [0.40; 0.46]
Degneh et al 2017	335	933	2.2%	0.3%	0.36 [0.33; 0.39]
Delafosse et al 2006	32	933	0.3%	0.3%	0.03 [0.02; 0.05]
de Maro Barbieri et al 2016	40	400	0.4%	0.3%	0.10 [0.07; 0.13]
Desquesnes and Gardiner 1993	870	3000	6.4%	0.3%	0.29 [0.27; 0.31]
Doko et al 1991	203	233	0.3%	0.3%	0.87 [0.82; 0.91]
Efrem et al 2010	24	568	0.2%	0.3%	0.04 [0.03; 0.06]
Enwezor et al 2011	203	964	1.7%	0.3%	0.21 [0.19; 0.24]
Enwezor et al 2012	14	395	0.1%	0.3%	0.04 [0.02; 0.06]
Ezeani et al 2008	50	132	0.3%	0.3%	0.38 [0.30; 0.47]
Ezeani et al 2008	58	132	0.3%	0.3%	0.44 [0.35; 0.53]
Fajinmi et al 2011	6	500	0.1%	0.2%	0.01 [0.00; 0.03]
Fentahun et al 2012	22	388	0.2%	0.3%	0.06 [0.04; 0.08]
Ganyo et al 2018	11	110	0.1%	0.3%	0.10 [0.05; 0.17]
Garcia et al 2005	1	15	0.0%	0.2%	0.07 [0.00; 0.32]
Garcia et al 2005	6	15	0.0%	0.2%	0.40 [0.16; 0.68]
Garcia et al 2005	3	15	0.0%	0.2%	0.20 [0.04; 0.48]
Girmay et al 2016	41	4870	0.4%	0.3%	0.01 [0.01; 0.01]
Gonzales et al 2007	25	202	0.2%	0.3%	0.12 [0.08; 0.18]
Gonzales et al 2007	55	202	0.4%	0.3%	0.27 [0.21; 0.34]
Gonzales et al 2007	5	209	0.1%	0.2%	0.02 [0.01; 0.05]
Gonzales et al 2007	37	209	0.3%	0.3%	0.18 [0.13; 0.24]
González and Meléndez 2007	158	400	1.0%	0.3%	0.40 [0.35; 0.44]
Guedes Junior et al 2008	54	65	0.1%	0.3%	0.83 [0.72; 0.91]
Guedes Junior et al 2008	175	181	0.1%	0.2%	0.97 [0.93; 0.99]
Guerra et al 2013	286	2053	2.6%	0.3%	0.14 [0.12; 0.16]
Haji et al 2014	1	56	0.0%	0.2%	0.02 [0.00; 0.10]
Haji et al 2014	3	58	0.0%	0.2%	0.05 [0.01; 0.14]
Haji et al 2014	3	60	0.0%	0.2%	0.05 [0.01; 0.14]
Haji et al 2014	0	59	0.0%	0.1%	0.00 [0.00; 0.06]
Haji et al 2014	1	62	0.0%	0.2%	0.02 [0.00; 0.09]
Haji et al 2015	25	56	0.1%	0.3%	0.45 [0.31; 0.59]
Haji et al 2015	29	58	0.2%	0.3%	0.50 [0.37; 0.63]
Haji et al 2015	4	59	0.0%	0.2%	0.07 [0.02; 0.16]
Haji et al 2015	12	62	0.1%	0.3%	0.19 [0.10; 0.31]
Haji et al 2015	10	60	0.1%	0.3%	0.17 [0.08; 0.29]
Hall et al 1983	61	1069	0.6%	0.3%	0.06 [0.04; 0.07]
Idehen et al 2018	13	361	0.1%	0.3%	0.04 [0.02; 0.06]
Jaimes−Dueñez et al 2019	0	710	0.0%	0.1%	0.00 [0.00; 0.01]
Kalu 1995	33	458	0.3%	0.3%	0.07 [0.05; 0.10]
Kalu et al 2001	9	268	0.1%	0.3%	0.03 [0.02; 0.06]
Kalu et al 2001	29	373	0.3%	0.3%	0.08 [0.05; 0.11]
Karimuribo et al 2011	8	171	0.1%	0.2%	0.05 [0.02; 0.09]
Kassaye 2015	11	599	0.1%	0.3%	0.02 [0.01; 0.03]
Kassaye 2015	11	599	0.1%	0.3%	0.02 [0.01; 0.03]
Kassian et al 2017	15	422	0.2%	0.3%	0.04 [0.02; 0.06]
Kassian et al 2017	88	422	0.7%	0.3%	0.21 [0.17; 0.25]
Kayang et al 1997	85	422	0.7%	0.3%	0.20 [0.16; 0.24]
Kidanemariam et al 2002	108	1008	1.0%	0.3%	0.11 [0.09; 0.13]
Kimaro et al 2018	7	96	0.1%	0.2%	0.07 [0.03; 0.14]
Kimaro et al 2018	2	96	0.0%	0.2%	0.02 [0.00; 0.07]
Kimaro et al 2018	4	96	0.0%	0.2%	0.04 [0.01; 0.10]
Kimaro et al 2018	8	96	0.1%	0.2%	0.08 [0.04; 0.16]
Kimaro et al 2018	2	96	0.0%	0.2%	0.02 [0.00; 0.07]
Kimaro et al 2018	1	96	0.0%	0.2%	0.01 [0.00; 0.06]
Kimaro et al 2018	2	96	0.0%	0.2%	0.02 [0.00; 0.07]
Kimaro et al 2018	8	96	0.1%	0.2%	0.08 [0.04; 0.16]
Kimaro et al 2018	5	96	0.0%	0.2%	0.05 [0.02; 0.12]
Kouadio et al 2014	1	45	0.0%	0.2%	0.02 [0.00; 0.12]
Kouadio et al 2014	6	59	0.1%	0.2%	0.10 [0.04; 0.21]
Kouadio et al 2014	3	60	0.0%	0.2%	0.05 [0.01; 0.14]
Kouadio et al 2014	5	60	0.0%	0.2%	0.08 [0.03; 0.18]
Kouadio et al 2014	23	67	0.2%	0.3%	0.34 [0.23; 0.47]
Kouadio et al 2014	2	72	0.0%	0.2%	0.03 [0.00; 0.10]
Laohasinnarong et al 2011	3	148	0.0%	0.2%	0.02 [0.00; 0.06]
Laohasinnarong et al 2015	0	46	0.0%	0.1%	0.00 [0.00; 0.08]
Laohasinnarong et al 2015	4	92	0.0%	0.2%	0.04 [0.01; 0.11]
Laohasinnarong et al 2015	1	105	0.0%	0.2%	0.01 [0.00; 0.05]
Lefrançois et al 1998	55	137	0.3%	0.3%	0.40 [0.32; 0.49]
Lelisa et al 2014	9	389	0.1%	0.3%	0.02 [0.01; 0.04]
Lema et al 2018	2	200	0.0%	0.2%	0.01 [0.00; 0.04]
Luckins and Mehlitz 1978	27	140	0.2%	0.3%	0.19 [0.13; 0.27]
Luckins and Mehlitz 1978	78	140	0.4%	0.3%	0.56 [0.47; 0.64]
Luckins and Mehlitz 1978	72	140	0.4%	0.3%	0.51 [0.43; 0.60]
Madruga et al 2006	86	150	0.4%	0.3%	0.57 [0.49; 0.65]
Madruga et al 2006	127	214	0.5%	0.3%	0.59 [0.52; 0.66]
Madruga et al 2006	141	501	1.1%	0.3%	0.28 [0.24; 0.32]
Madruga et al 2006	359	516	1.1%	0.3%	0.70 [0.65; 0.74]
Madruga et al 2006	394	548	1.2%	0.3%	0.72 [0.68; 0.76]
Madruga et al 2006	298	579	1.5%	0.3%	0.51 [0.47; 0.56]
Maganga et al 2017	1	14	0.0%	0.2%	0.07 [0.00; 0.34]
Maganga et al 2017	4	100	0.0%	0.2%	0.04 [0.01; 0.10]
Maganga et al 2017	11	110	0.1%	0.3%	0.10 [0.05; 0.17]
Magona et al 1999	0	4	0.0%	0.1%	0.00 [0.00; 0.60]
Magona et al 1999	2	6	0.0%	0.2%	0.33 [0.04; 0.78]
Magona et al 1999	1	49	0.0%	0.2%	0.02 [0.00; 0.11]
Magona et al 2000	41	800	0.4%	0.3%	0.05 [0.04; 0.07]
Magona et al 2003	13	295	0.1%	0.3%	0.04 [0.02; 0.07]
Magona et al 2003	12	295	0.1%	0.3%	0.04 [0.02; 0.07]
Magona et al 2004	16	260	0.2%	0.3%	0.06 [0.04; 0.10]
Magona et al 2004	66	1215	0.6%	0.3%	0.05 [0.04; 0.07]
Magona et al 2005	4	99	0.0%	0.2%	0.04 [0.01; 0.10]
Magona et al 2005	7	100	0.1%	0.2%	0.07 [0.03; 0.14]
Magona et al 2005	4	102	0.0%	0.2%	0.04 [0.01; 0.10]
Magona et al 2005	5	103	0.0%	0.2%	0.05 [0.02; 0.11]
Magona et al 2005	3	107	0.0%	0.2%	0.03 [0.01; 0.08]
Magona et al 2005	6	107	0.1%	0.2%	0.06 [0.02; 0.12]
Magona et al 2005	3	108	0.0%	0.2%	0.03 [0.01; 0.08]
Magona et al 2005	10	113	0.1%	0.3%	0.09 [0.04; 0.16]
Magona et al 2005	3	140	0.0%	0.2%	0.02 [0.00; 0.06]
Magona et al 2005	9	163	0.1%	0.3%	0.06 [0.03; 0.10]
Magona et al 2005	18	219	0.2%	0.3%	0.08 [0.05; 0.13]
Magona et al 2005	7	252	0.1%	0.2%	0.03 [0.01; 0.06]
Magona et al 2005	5	279	0.1%	0.2%	0.02 [0.01; 0.04]
Magona et al 2005	0	100	0.0%	0.1%	0.00 [0.00; 0.04]
Magona et al 2008	40	401	0.4%	0.3%	0.10 [0.07; 0.13]
Magona et al 2011	2	37	0.0%	0.2%	0.05 [0.01; 0.18]
Magona et al 2011	4	100	0.0%	0.2%	0.04 [0.01; 0.10]
Magona et al 2011	18	100	0.2%	0.3%	0.18 [0.11; 0.27]
Magona et al 2011	1	100	0.0%	0.2%	0.01 [0.00; 0.05]
Magona et al 2011	4	100	0.0%	0.2%	0.04 [0.01; 0.10]
Magona et al 2011	2	100	0.0%	0.2%	0.02 [0.00; 0.07]
Magona et al 2011	4	100	0.0%	0.2%	0.04 [0.01; 0.10]
Magona et al 2011	3	100	0.0%	0.2%	0.03 [0.01; 0.09]
Magona et al 2011	1	100	0.0%	0.2%	0.01 [0.00; 0.05]
Magona et al 2011	5	100	0.0%	0.2%	0.05 [0.02; 0.11]
Magona et al 2011	3	100	0.0%	0.2%	0.03 [0.01; 0.09]
Magona et al 2011	6	100	0.1%	0.2%	0.06 [0.02; 0.13]
Magona et al 2011	6	100	0.1%	0.2%	0.06 [0.02; 0.13]
Magona et al 2011	0	100	0.0%	0.1%	0.00 [0.00; 0.04]
Magona et al 2011	4	100	0.0%	0.2%	0.04 [0.01; 0.10]
Magona et al 2011	3	100	0.0%	0.2%	0.03 [0.01; 0.09]
Magona et al 2011	6	125	0.1%	0.2%	0.05 [0.02; 0.10]
Magona et al 2011	12	200	0.1%	0.3%	0.06 [0.03; 0.10]
Magona et al 2011	7	200	0.1%	0.2%	0.04 [0.01; 0.07]
Magona et al 2011	11	300	0.1%	0.3%	0.04 [0.02; 0.06]
Makumyaviri and Ngarambe 1997	39	180	0.3%	0.3%	0.22 [0.16; 0.28]
Mamabolo et al 2009	1	38	0.0%	0.2%	0.03 [0.00; 0.14]
Mamabolo et al 2009	5	45	0.0%	0.2%	0.11 [0.04; 0.24]
Mamabolo et al 2009	24	60	0.1%	0.3%	0.40 [0.28; 0.53]
Mamoudou et al 2016	57	866	0.6%	0.3%	0.07 [0.05; 0.08]
Manuel Tafur et al 2002	27	270	0.3%	0.3%	0.10 [0.07; 0.14]
Mbahin et al 2013	8	35	0.1%	0.2%	0.23 [0.10; 0.40]
Mbahin et al 2013	7	48	0.1%	0.2%	0.15 [0.06; 0.28]
Mbahin et al 2013	13	51	0.1%	0.3%	0.25 [0.14; 0.40]
Mbahin et al 2013	1	54	0.0%	0.2%	0.02 [0.00; 0.10]
Mbahin et al 2013	13	56	0.1%	0.3%	0.23 [0.13; 0.36]
Mbahin et al 2013	2	59	0.0%	0.2%	0.03 [0.00; 0.12]
Mbahin et al 2013	7	61	0.1%	0.2%	0.11 [0.05; 0.22]
Mbahin et al 2013	6	64	0.1%	0.2%	0.09 [0.04; 0.19]
Mbahin et al 2013	16	66	0.1%	0.3%	0.24 [0.15; 0.36]
Mbahin et al 2013	14	90	0.1%	0.3%	0.16 [0.09; 0.25]
Mbewe et al 2015	1	115	0.0%	0.2%	0.01 [0.00; 0.05]
Mbewe et al 2015	1	152	0.0%	0.2%	0.01 [0.00; 0.04]
Mehlitz 1979	11	42	0.1%	0.3%	0.26 [0.14; 0.42]
Mehlitz 1979	10	42	0.1%	0.2%	0.24 [0.12; 0.39]
Mehlitz 1979	36	44	0.1%	0.2%	0.82 [0.67; 0.92]
Mehlitz 1979	38	44	0.1%	0.2%	0.86 [0.73; 0.95]
Mehlitz 1979	31	54	0.1%	0.3%	0.57 [0.43; 0.71]
Mehlitz 1979	24	54	0.1%	0.3%	0.44 [0.31; 0.59]
Mekata et al 2009	4	104	0.0%	0.2%	0.04 [0.01; 0.10]
Mekata et al 2009	5	510	0.1%	0.2%	0.01 [0.00; 0.02]
Mekuria and Gadissa 2011	5	98	0.0%	0.2%	0.05 [0.02; 0.12]
Mekuria and Gadissa 2011	3	135	0.0%	0.2%	0.02 [0.00; 0.06]
Mekuria and Gadissa 2011	2	307	0.0%	0.2%	0.01 [0.00; 0.02]
Melese et al 2017	1	125	0.0%	0.2%	0.01 [0.00; 0.04]
Melese et al 2017	5	150	0.1%	0.2%	0.03 [0.01; 0.08]
Mihret and Mamo 2007	39	1120	0.4%	0.3%	0.03 [0.02; 0.05]
Mihret and Mamo 2007	113	1120	1.1%	0.3%	0.10 [0.08; 0.12]
Mihret and Mamo 2007	97	1120	0.9%	0.3%	0.09 [0.07; 0.10]
Moti et al 2014	23	411	0.2%	0.3%	0.06 [0.04; 0.08]
Moti et al 2014	2	411	0.0%	0.2%	0.00 [0.00; 0.02]
Moti et al 2014	23	411	0.2%	0.3%	0.06 [0.04; 0.08]
Mugittu et al 2001	1	50	0.0%	0.2%	0.02 [0.00; 0.11]
Mugittu et al 2001	0	62	0.0%	0.1%	0.00 [0.00; 0.06]
Mugittu et al 2001	9	278	0.1%	0.3%	0.03 [0.01; 0.06]
Muhanguzi et al 2014	813	6053	7.3%	0.3%	0.13 [0.13; 0.14]
Muhanguzi et al 2017	11	46	0.1%	0.3%	0.24 [0.13; 0.39]
Muhanguzi et al 2017	0	50	0.0%	0.1%	0.00 [0.00; 0.07]
Muhanguzi et al 2017	0	50	0.0%	0.1%	0.00 [0.00; 0.07]
Muhanguzi et al 2017	0	56	0.0%	0.1%	0.00 [0.00; 0.06]
Muhanguzi et al 2017	0	56	0.0%	0.1%	0.00 [0.00; 0.06]
Muhanguzi et al 2017	0	56	0.0%	0.1%	0.00 [0.00; 0.06]
Muhanguzi et al 2017	0	56	0.0%	0.1%	0.00 [0.00; 0.06]
Muhanguzi et al 2017	0	56	0.0%	0.1%	0.00 [0.00; 0.06]
Muhanguzi et al 2017	0	56	0.0%	0.1%	0.00 [0.00; 0.06]
Muhanguzi et al 2017	1	56	0.0%	0.2%	0.02 [0.00; 0.10]
Muhanguzi et al 2017	5	56	0.0%	0.2%	0.09 [0.03; 0.20]
Muhanguzi et al 2017	8	56	0.1%	0.2%	0.14 [0.06; 0.26]
Muhanguzi et al 2017	10	56	0.1%	0.3%	0.18 [0.09; 0.30]
Muhanguzi et al 2017	0	56	0.0%	0.1%	0.00 [0.00; 0.06]
Muhanguzi et al 2017	0	56	0.0%	0.1%	0.00 [0.00; 0.06]
Muhanguzi et al 2017	9	56	0.1%	0.2%	0.16 [0.08; 0.28]
Muhanguzi et al 2017	0	60	0.0%	0.1%	0.00 [0.00; 0.06]
Muhanguzi et al 2017	0	60	0.0%	0.1%	0.00 [0.00; 0.06]
Muhanguzi et al 2017	32	60	0.2%	0.3%	0.53 [0.40; 0.66]
Muhanguzi et al 2017	0	60	0.0%	0.1%	0.00 [0.00; 0.06]
Muhanguzi et al 2017	5	60	0.0%	0.2%	0.08 [0.03; 0.18]
Muhanguzi et al 2017	0	60	0.0%	0.1%	0.00 [0.00; 0.06]
Muhanguzi et al 2017	0	60	0.0%	0.1%	0.00 [0.00; 0.06]
Muhanguzi et al 2017	0	60	0.0%	0.1%	0.00 [0.00; 0.06]
Muhanguzi et al 2017	0	60	0.0%	0.1%	0.00 [0.00; 0.06]
Muhanguzi et al 2017	0	60	0.0%	0.1%	0.00 [0.00; 0.06]
Muhanguzi et al 2017	7	60	0.1%	0.2%	0.12 [0.05; 0.23]
Muhanguzi et al 2017	8	60	0.1%	0.2%	0.13 [0.06; 0.25]
Muhanguzi et al 2017	0	60	0.0%	0.1%	0.00 [0.00; 0.06]
Muhanguzi et al 2017	0	60	0.0%	0.1%	0.00 [0.00; 0.06]
Muhanguzi et al 2017	11	60	0.1%	0.3%	0.18 [0.10; 0.30]
Muhanguzi et al 2017	0	62	0.0%	0.1%	0.00 [0.00; 0.06]
Muhanguzi et al 2017	0	62	0.0%	0.1%	0.00 [0.00; 0.06]
Muhanguzi et al 2017	15	82	0.1%	0.3%	0.18 [0.11; 0.28]
Mulaw et al 2011	10	384	0.1%	0.3%	0.03 [0.01; 0.05]
Mungube et al 2012	2	96	0.0%	0.2%	0.02 [0.00; 0.07]
Mungube et al 2012	4	100	0.0%	0.2%	0.04 [0.01; 0.10]
Mungube et al 2012	2	100	0.0%	0.2%	0.02 [0.00; 0.07]
Mungube et al 2012	6	100	0.1%	0.2%	0.06 [0.02; 0.13]
Mungube et al 2012	2	100	0.0%	0.2%	0.02 [0.00; 0.07]
Mungube et al 2012	4	100	0.0%	0.2%	0.04 [0.01; 0.10]
Mungube et al 2012	4	100	0.0%	0.2%	0.04 [0.01; 0.10]
Mungube et al 2012	10	100	0.1%	0.3%	0.10 [0.05; 0.18]
Musinguzi et al 2016	0	84	0.0%	0.1%	0.00 [0.00; 0.04]
Musinguzi et al 2016	38	96	0.2%	0.3%	0.40 [0.30; 0.50]
Musinguzi et al 2016	46	292	0.4%	0.3%	0.16 [0.12; 0.20]
Mwambu 1969	4	63	0.0%	0.2%	0.06 [0.02; 0.15]
Mwambu 1969	0	168	0.0%	0.1%	0.00 [0.00; 0.02]
Mwambu 1969	0	175	0.0%	0.1%	0.00 [0.00; 0.02]
Mwambu 1969	4	223	0.0%	0.2%	0.02 [0.00; 0.05]
Mwambu 1969	7	260	0.1%	0.2%	0.03 [0.01; 0.05]
Mwambu 1969	0	270	0.0%	0.1%	0.00 [0.00; 0.01]
Mwambu 1969	8	290	0.1%	0.2%	0.03 [0.01; 0.05]
Mwambu 1969	7	317	0.1%	0.2%	0.02 [0.01; 0.04]
Mwambu 1969	8	370	0.1%	0.3%	0.02 [0.01; 0.04]
Mwambu 1969	4	390	0.0%	0.2%	0.01 [0.00; 0.03]
Mwambu 1969	5	1306	0.1%	0.2%	0.00 [0.00; 0.01]
Mwambu and Mayende 1973	23	798	0.2%	0.3%	0.03 [0.02; 0.04]
Mwambu and Mayende 1973	0	798	0.0%	0.1%	0.00 [0.00; 0.00]
N'Djetchi et al 2017	3	87	0.0%	0.2%	0.03 [0.01; 0.10]
Nakayima et al 2012	37	146	0.3%	0.3%	0.25 [0.19; 0.33]
Nakayima et al 2013	32	100	0.2%	0.3%	0.32 [0.23; 0.42]
Nantulya et al 1992	77	301	0.6%	0.3%	0.26 [0.21; 0.31]
Nantulya et al 1992	33	301	0.3%	0.3%	0.11 [0.08; 0.15]
Ngomtcho et al 2017	0	35	0.0%	0.1%	0.00 [0.00; 0.10]
Ngomtcho et al 2017	1	100	0.0%	0.2%	0.01 [0.00; 0.05]
Ngomtcho et al 2017	4	120	0.0%	0.2%	0.03 [0.01; 0.08]
Ngomtcho et al 2017	1	137	0.0%	0.2%	0.01 [0.00; 0.04]
Nonga and Kambarage 2009	9	691	0.1%	0.3%	0.01 [0.01; 0.02]
Ocaido et al 2005	27	400	0.3%	0.3%	0.07 [0.04; 0.10]
Ode et al 2017	5	200	0.1%	0.2%	0.02 [0.01; 0.06]
Odeniran et al 2019	86	745	0.8%	0.3%	0.12 [0.09; 0.14]
Odongo et al 2016	70	358	0.6%	0.3%	0.20 [0.16; 0.24]
Odongo et al 2016	60	358	0.5%	0.3%	0.17 [0.13; 0.21]
Pagabeleguem et al 2012	6	96	0.1%	0.2%	0.06 [0.02; 0.13]
Pagabeleguem et al 2012	44	96	0.2%	0.3%	0.46 [0.36; 0.56]
Pagabeleguem et al 2012	1	185	0.0%	0.2%	0.01 [0.00; 0.03]
Pagabeleguem et al 2012	32	185	0.3%	0.3%	0.17 [0.12; 0.24]
Pagabeleguem et al 2012	40	364	0.4%	0.3%	0.11 [0.08; 0.15]
Pagabeleguem et al 2012	124	364	0.9%	0.3%	0.34 [0.29; 0.39]
Pagabeleguem et al 2012	0	396	0.0%	0.1%	0.00 [0.00; 0.01]
Pagabeleguem et al 2012	36	396	0.3%	0.3%	0.09 [0.06; 0.12]
Paoletta et al 2018	2	38	0.0%	0.2%	0.05 [0.01; 0.18]
Paoletta et al 2018	13	54	0.1%	0.3%	0.24 [0.13; 0.38]
Paoletta et al 2018	1	94	0.0%	0.2%	0.01 [0.00; 0.06]
Patricia Quispe et al 2003	1	24	0.0%	0.2%	0.04 [0.00; 0.21]
Patricia Quispe et al 2003	0	24	0.0%	0.1%	0.00 [0.00; 0.14]
Patricia Quispe et al 2003	0	26	0.0%	0.1%	0.00 [0.00; 0.13]
Patricia Quispe et al 2003	1	26	0.0%	0.2%	0.04 [0.00; 0.20]
Patricia Quispe et al 2003	0	26	0.0%	0.1%	0.00 [0.00; 0.13]
Patricia Quispe et al 2003	1	26	0.0%	0.2%	0.04 [0.00; 0.20]
Patricia Quispe et al 2003	62	205	0.5%	0.3%	0.30 [0.24; 0.37]
Patricia Quispe et al 2003	16	205	0.2%	0.3%	0.08 [0.05; 0.12]
Rahman 2005	4	20	0.0%	0.2%	0.20 [0.06; 0.44]
Rahman 2005	10	32	0.1%	0.2%	0.31 [0.16; 0.50]
Rahman 2005	1	108	0.0%	0.2%	0.01 [0.00; 0.05]
Rahman 2005	22	110	0.2%	0.3%	0.20 [0.13; 0.29]
Rahman 2005	6	120	0.1%	0.2%	0.05 [0.02; 0.11]
Rahman 2005	6	140	0.1%	0.2%	0.04 [0.02; 0.09]
Rahman 2005	6	150	0.1%	0.2%	0.04 [0.01; 0.09]
Rahman 2005	9	150	0.1%	0.3%	0.06 [0.03; 0.11]
Rahman 2005	8	160	0.1%	0.2%	0.05 [0.02; 0.10]
Rahman 2005	18	180	0.2%	0.3%	0.10 [0.06; 0.15]
Rahman 2005	2	200	0.0%	0.2%	0.01 [0.00; 0.04]
Rahman 2005	4	200	0.0%	0.2%	0.02 [0.01; 0.05]
Rahman 2005	12	200	0.1%	0.3%	0.06 [0.03; 0.10]
Rahman 2005	81	300	0.6%	0.3%	0.27 [0.22; 0.32]
Rahman 2005	2	400	0.0%	0.2%	0.00 [0.00; 0.02]
Ramirez−Iglesias et al 2017	0	38	0.0%	0.1%	0.00 [0.00; 0.09]
Ramirez−Iglesias et al 2017	9	47	0.1%	0.2%	0.19 [0.09; 0.33]
Ravel et al 2015	0	104	0.0%	0.1%	0.00 [0.00; 0.03]
Regassa et al 2012	3	145	0.0%	0.2%	0.02 [0.00; 0.06]
Regassa et al 2012	49	145	0.3%	0.3%	0.34 [0.26; 0.42]
Regassa et al 2012	1	199	0.0%	0.2%	0.01 [0.00; 0.03]
Regassa et al 2012	29	199	0.3%	0.3%	0.15 [0.10; 0.20]
Regassa et al 2012	21	212	0.2%	0.3%	0.10 [0.06; 0.15]
Regassa et al 2012	2	212	0.0%	0.2%	0.01 [0.00; 0.03]
Regassa et al 2012	42	212	0.4%	0.3%	0.20 [0.15; 0.26]
Regassa et al 2012	67	212	0.5%	0.3%	0.32 [0.25; 0.38]
Regassa et al 2012	15	373	0.1%	0.3%	0.04 [0.02; 0.07]
Regassa et al 2012	68	373	0.6%	0.3%	0.18 [0.14; 0.23]
Regassa et al 2012	11	383	0.1%	0.3%	0.03 [0.01; 0.05]
Regassa et al 2012	63	383	0.5%	0.3%	0.16 [0.13; 0.21]
Reis et al 2019	178	971	1.5%	0.3%	0.18 [0.16; 0.21]
Robson and Rickman 1972	38	265	0.3%	0.3%	0.14 [0.10; 0.19]
Robson and Rickman 1972	26	265	0.2%	0.3%	0.10 [0.07; 0.14]
Robson and Rickman 1972	36	265	0.3%	0.3%	0.14 [0.10; 0.18]
Salas et al 2017	12	326	0.1%	0.3%	0.04 [0.02; 0.06]
Salas et al 2017	1	337	0.0%	0.2%	0.00 [0.00; 0.02]
Salas et al 2017	1	337	0.0%	0.2%	0.00 [0.00; 0.02]
Samdi et al 2010	51	68	0.1%	0.3%	0.75 [0.63; 0.85]
Samdi et al 2011	2	634	0.0%	0.2%	0.00 [0.00; 0.01]
Seck et al 2010	23	1141	0.2%	0.3%	0.02 [0.01; 0.03]
Seck et al 2010	327	1141	2.4%	0.3%	0.29 [0.26; 0.31]
Seck et al 2010	8	1141	0.1%	0.3%	0.01 [0.00; 0.01]
Sharma et al 2001	33	1809	0.3%	0.3%	0.02 [0.01; 0.03]
Sheferaw et al 2016	44	1838	0.4%	0.3%	0.02 [0.02; 0.03]
Silbermayr et al 2013	34	368	0.3%	0.3%	0.09 [0.06; 0.13]
Silbermayr et al 2013	33	368	0.3%	0.3%	0.09 [0.06; 0.12]
Silbermayr et al 2013	34	368	0.3%	0.3%	0.09 [0.06; 0.13]
Simukoko et al 2007	16	734	0.2%	0.3%	0.02 [0.01; 0.04]
Simukoko et al 2007	18	734	0.2%	0.3%	0.02 [0.01; 0.04]
Simwango et al 2017	100	1002	0.9%	0.3%	0.10 [0.08; 0.12]
Sinshaw et al 2006	6	133	0.1%	0.2%	0.05 [0.02; 0.10]
Sinshaw et al 2006	7	175	0.1%	0.2%	0.04 [0.02; 0.08]
Sinshaw et al 2006	99	1201	0.9%	0.3%	0.08 [0.07; 0.10]
Sinyangwe et al 2004	5	1597	0.1%	0.2%	0.00 [0.00; 0.01]
Snak et al 2018	0	40	0.0%	0.1%	0.00 [0.00; 0.09]
Snak et al 2018	0	60	0.0%	0.1%	0.00 [0.00; 0.06]
Solano et al 1999	3	260	0.0%	0.2%	0.01 [0.00; 0.03]
Solano et al 1999	7	260	0.1%	0.2%	0.03 [0.01; 0.05]
Sow et al 2013	21	2002	0.2%	0.3%	0.01 [0.01; 0.02]
Specht 2008	6	52	0.1%	0.2%	0.12 [0.04; 0.23]
Specht 2008	5	71	0.0%	0.2%	0.07 [0.02; 0.16]
Specht 2008	3	87	0.0%	0.2%	0.03 [0.01; 0.10]
Specht 2008	9	124	0.1%	0.3%	0.07 [0.03; 0.13]
Specht 2008	20	173	0.2%	0.3%	0.12 [0.07; 0.17]
Specht 2008	23	218	0.2%	0.3%	0.11 [0.07; 0.15]
Specht 2008	14	245	0.1%	0.3%	0.06 [0.03; 0.09]
Specht 2008	51	247	0.4%	0.3%	0.21 [0.16; 0.26]
Specht 2008	34	258	0.3%	0.3%	0.13 [0.09; 0.18]
Specht 2008	40	258	0.4%	0.3%	0.16 [0.11; 0.21]
Specht 2008	28	268	0.3%	0.3%	0.10 [0.07; 0.15]
Specht 2008	21	270	0.2%	0.3%	0.08 [0.05; 0.12]
Specht 2008	15	272	0.1%	0.3%	0.06 [0.03; 0.09]
Specht 2008	11	278	0.1%	0.3%	0.04 [0.02; 0.07]
Specht 2008	17	282	0.2%	0.3%	0.06 [0.04; 0.09]
Specht 2008	9	292	0.1%	0.3%	0.03 [0.01; 0.06]
Specht 2008	56	330	0.5%	0.3%	0.17 [0.13; 0.21]
Specht 2008	16	331	0.2%	0.3%	0.05 [0.03; 0.08]
Specht 2008	21	335	0.2%	0.3%	0.06 [0.04; 0.09]
Specht 2008	25	343	0.2%	0.3%	0.07 [0.05; 0.11]
Specht 2008	7	347	0.1%	0.2%	0.02 [0.01; 0.04]
Specht 2008	33	359	0.3%	0.3%	0.09 [0.06; 0.13]
Specht 2008	27	363	0.3%	0.3%	0.07 [0.05; 0.11]
Specht 2008	23	367	0.2%	0.3%	0.06 [0.04; 0.09]
Specht 2008	28	381	0.3%	0.3%	0.07 [0.05; 0.10]
Specht 2008	19	388	0.2%	0.3%	0.05 [0.03; 0.08]
Specht 2008	17	473	0.2%	0.3%	0.04 [0.02; 0.06]
Specht 2008	48	748	0.5%	0.3%	0.06 [0.05; 0.08]
Specht 2008	49	828	0.5%	0.3%	0.06 [0.04; 0.08]
Specht 2008	113	930	1.0%	0.3%	0.12 [0.10; 0.14]
Specht 2008	104	1657	1.0%	0.3%	0.06 [0.05; 0.08]
Specht 2008	40	1696	0.4%	0.3%	0.02 [0.02; 0.03]
Specht 2008	148	1800	1.4%	0.3%	0.08 [0.07; 0.10]
Specht 2008	165	1824	1.6%	0.3%	0.09 [0.08; 0.10]
Suh et al 2017	6	770	0.1%	0.2%	0.01 [0.00; 0.02]
Swai and Kaaya 2012	0	30	0.0%	0.1%	0.00 [0.00; 0.12]
Swai and Kaaya 2012	2	34	0.0%	0.2%	0.06 [0.01; 0.20]
Swai and Kaaya 2012	4	85	0.0%	0.2%	0.05 [0.01; 0.12]
Swai and Kaaya 2012	2	90	0.0%	0.2%	0.02 [0.00; 0.08]
Tadesse and Tsegaye 2010	1	61	0.0%	0.2%	0.02 [0.00; 0.09]
Tadesse and Tsegaye 2010	1	70	0.0%	0.2%	0.01 [0.00; 0.08]
Tafese et al 2012	9	386	0.1%	0.3%	0.02 [0.01; 0.04]
Takeet et al 2013	18	411	0.2%	0.3%	0.04 [0.03; 0.07]
Takeet et al 2013	21	411	0.2%	0.3%	0.05 [0.03; 0.08]
Tasew and Duguma 2012	2	161	0.0%	0.2%	0.01 [0.00; 0.04]
Tasew and Duguma 2012	4	372	0.0%	0.2%	0.01 [0.00; 0.03]
Tasew and Duguma 2012	6	599	0.1%	0.2%	0.01 [0.00; 0.02]
Tchamdja et al 2017	11	156	0.1%	0.3%	0.07 [0.04; 0.12]
Tchamdja et al 2017	16	198	0.2%	0.3%	0.08 [0.05; 0.13]
Terefe et al 2015	10	409	0.1%	0.3%	0.02 [0.01; 0.04]
Tesfaheywet et al 2012	2	120	0.0%	0.2%	0.02 [0.00; 0.06]
Tesfaheywet et al 2012	0	124	0.0%	0.1%	0.00 [0.00; 0.03]
Tesfaheywet et al 2012	6	140	0.1%	0.2%	0.04 [0.02; 0.09]
Tesfaye and Ibrahim 2017	4	50	0.0%	0.2%	0.08 [0.02; 0.19]
Tesfaye and Ibrahim 2017	3	60	0.0%	0.2%	0.05 [0.01; 0.14]
Tesfaye and Ibrahim 2017	9	200	0.1%	0.3%	0.04 [0.02; 0.08]
Thumbi et al2010	15	44	0.1%	0.3%	0.34 [0.20; 0.50]
Thumbi et al2010	22	59	0.1%	0.3%	0.37 [0.25; 0.51]
Ukpai and Obasi2017	8	115	0.1%	0.2%	0.07 [0.03; 0.13]
Van den Bossche et al 1999	15	1481	0.2%	0.3%	0.01 [0.01; 0.02]
Van den Bossche et al 2000	9	9309	0.1%	0.3%	0.00 [0.00; 0.00]
Waiswa and Katunguka−Rwakishaya 2004	31	648	0.3%	0.3%	0.05 [0.03; 0.07]
Waiswa and Katunguka−Rwakishaya 2004	40	661	0.4%	0.3%	0.06 [0.04; 0.08]
Wijers 1974	31	307	0.3%	0.3%	0.10 [0.07; 0.14]
Yesufu and Mshelbwala 1973	12	20	0.0%	0.2%	0.60 [0.36; 0.81]
Yesufu and Mshelbwala 1973	9	25	0.1%	0.2%	0.36 [0.18; 0.57]
Yesufu and Mshelbwala 1973	20	30	0.1%	0.2%	0.67 [0.47; 0.83]
Zanatto et al 2019	7	10	0.0%	0.2%	0.70 [0.35; 0.93]
Zanatto et al 2019	11	20	0.1%	0.2%	0.55 [0.32; 0.77]
Zanatto et al 2019	11	32	0.1%	0.2%	0.34 [0.19; 0.53]
Zanatto et al 2019	22	40	0.1%	0.3%	0.55 [0.38; 0.71]
**Total (fixed effect, 95% CI)**		**150928**	**100.0%**	**--**	**0.15 [0.14; 0.15]**
**Total (random effects, 95% CI)**			**--**	**100.0%**	**0.06 [0.06; 0.07]**

Heterogeneity: $Tau^2 = 1.4098$; $Chi^2 = 13756.10$, df = 442 (P = 0); $I^2 = 97\%$

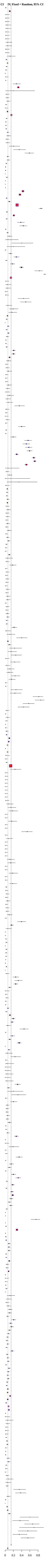

Dog

Study	Events	Total	Weight (fixed)	Weight (random)	IV, Fixed + Random, 95% CI
Joshua 1989	0	192	10.5%	10.6%	0.00 [0.00; 0.02]
Magona et al 1999	0	1	7.9%	8.1%	0.00 [0.00; 0.98]
Magona et al 1999	0	8	10.0%	10.1%	0.00 [0.00; 0.37]
Magona et al 1999	0	9	10.0%	10.2%	0.00 [0.00; 0.34]
Magona et al 1999	0	12	10.2%	10.3%	0.00 [0.00; 0.26]
Makumyaviri et al 1989	0	6	9.8%	9.9%	0.00 [0.00; 0.46]
Makumyaviri et al 1989	0	29	10.4%	10.5%	0.00 [0.00; 0.12]
Nimpaye et al 2011	1	37	20.6%	19.7%	0.03 [0.00; 0.14]
Ravel et al 2015	0	152	10.5%	10.6%	0.00 [0.00; 0.02]
**Total (fixed effect, 95% CI)**		**446**	**100.0%**	**--**	**0.03 [0.01; 0.06]**
**Total (random effects, 95% CI)**			**--**	**100.0%**	**0.03 [0.01; 0.06]**

Heterogeneity: $Tau^2 = 0.1212$; $Chi^2 = 8.50$, df = 8 (P = 0.39); $I^2 = 6\%$

IV, Fixed + Random, 95% CI

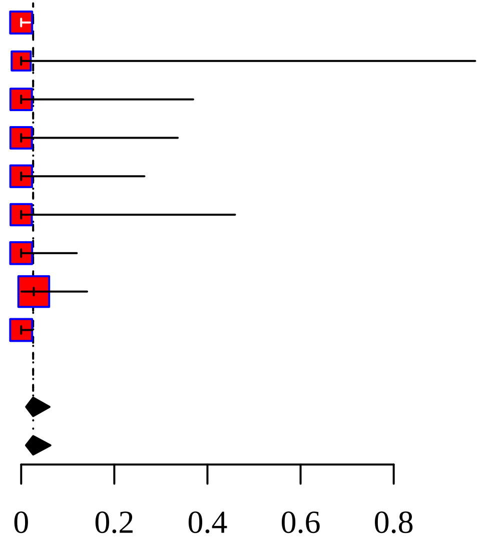

Equine

Study	Events	Total	Weight (fixed)	Weight (random)	IV, Fixed + Random, 95% CI
Abebe and Wolde 2010	0	52	0.5%	1.5%	0.00 [0.00; 0.07]
Abebe and Wolde 2010	4	67	3.6%	2.0%	0.06 [0.02; 0.15]
Abebe and Wolde 2010	0	81	0.5%	1.5%	0.00 [0.00; 0.04]
Abebe and Wolde 2010	0	91	0.5%	1.5%	0.00 [0.00; 0.04]
Abebe and Wolde 2010	0	95	0.5%	1.5%	0.00 [0.00; 0.04]
Birhanu et al 2015	3	38	2.7%	1.9%	0.08 [0.02; 0.21]
Ehizibolo et al 2012	1	243	1.0%	1.7%	0.00 [0.00; 0.02]
Eyob et al 2011	2	25	1.8%	1.9%	0.08 [0.01; 0.26]
Eyob et al 2011	2	85	1.9%	1.9%	0.02 [0.00; 0.08]
Eyob et al 2011	2	104	1.9%	1.9%	0.02 [0.00; 0.07]
Garcia et al 2005	3	6	1.5%	1.8%	0.50 [0.12; 0.88]
Mekibib et al 2010	1	194	1.0%	1.7%	0.01 [0.00; 0.03]
Mekibib et al 2010	2	206	1.9%	1.9%	0.01 [0.00; 0.03]
Mekuria et al 2010	0	20	0.5%	1.5%	0.00 [0.00; 0.17]
Mekuria et al 2010	0	60	0.5%	1.5%	0.00 [0.00; 0.06]
Mekuria et al 2010	6	214	5.6%	2.0%	0.03 [0.01; 0.06]
Muhanguzi et al 2017	0	2	0.4%	1.4%	0.00 [0.00; 0.84]
Muhanguzi et al 2017	0	2	0.4%	1.4%	0.00 [0.00; 0.84]
Muhanguzi et al 2017	0	2	0.4%	1.4%	0.00 [0.00; 0.84]
Muhanguzi et al 2017	0	3	0.4%	1.4%	0.00 [0.00; 0.71]
Muhanguzi et al 2017	0	4	0.4%	1.5%	0.00 [0.00; 0.60]
Muhanguzi et al 2017	0	4	0.4%	1.5%	0.00 [0.00; 0.60]
Muhanguzi et al 2017	0	4	0.4%	1.5%	0.00 [0.00; 0.60]
Muhanguzi et al 2017	0	4	0.4%	1.5%	0.00 [0.00; 0.60]
Muhanguzi et al 2017	0	5	0.4%	1.5%	0.00 [0.00; 0.52]
Muhanguzi et al 2017	0	5	0.4%	1.5%	0.00 [0.00; 0.52]
Muhanguzi et al 2017	0	6	0.4%	1.5%	0.00 [0.00; 0.46]
Muhanguzi et al 2017	0	6	0.4%	1.5%	0.00 [0.00; 0.46]
Muhanguzi et al 2017	0	8	0.5%	1.5%	0.00 [0.00; 0.37]
Muhanguzi et al 2017	0	16	0.5%	1.5%	0.00 [0.00; 0.21]
Pinchbeck et al 2008	45	58	9.8%	2.0%	0.78 [0.65; 0.87]
Pinchbeck et al 2008	165	183	15.7%	2.0%	0.90 [0.85; 0.94]
Ravel et al 2015	0	23	0.5%	1.5%	0.00 [0.00; 0.15]
Ravel et al 2015	0	63	0.5%	1.5%	0.00 [0.00; 0.06]
Rodrigues et al 2015	30	180	24.2%	2.0%	0.17 [0.12; 0.23]
Rodrigues et al 2015	0	180	0.5%	1.5%	0.00 [0.00; 0.02]
Sabir et al 2018	0	100	0.5%	1.5%	0.00 [0.00; 0.04]
Sabir et al 2018	0	100	0.5%	1.5%	0.00 [0.00; 0.04]
Sabir et al 2018	0	100	0.5%	1.5%	0.00 [0.00; 0.04]
Salim et al 2014	0	4	0.4%	1.5%	0.00 [0.00; 0.60]
Salim et al 2014	0	5	0.4%	1.5%	0.00 [0.00; 0.52]
Salim et al 2014	1	9	0.9%	1.7%	0.11 [0.00; 0.48]
Salim et al 2014	1	10	0.9%	1.7%	0.10 [0.00; 0.45]
Salim et al 2014	0	14	0.5%	1.5%	0.00 [0.00; 0.23]
Salim et al 2014	0	15	0.5%	1.5%	0.00 [0.00; 0.22]
Salim et al 2014	3	16	2.4%	1.9%	0.19 [0.04; 0.46]
Salim et al 2014	0	17	0.5%	1.5%	0.00 [0.00; 0.20]
Salim et al 2014	2	17	1.7%	1.9%	0.12 [0.01; 0.36]
Salim et al 2014	0	18	0.5%	1.5%	0.00 [0.00; 0.19]
Salim et al 2014	0	18	0.5%	1.5%	0.00 [0.00; 0.19]
Salim et al 2014	0	19	0.5%	1.5%	0.00 [0.00; 0.18]
Salim et al 2014	0	23	0.5%	1.5%	0.00 [0.00; 0.15]
Salim et al 2014	1	24	0.9%	1.7%	0.04 [0.00; 0.21]
Salim et al 2014	0	25	0.5%	1.5%	0.00 [0.00; 0.14]
Salim et al 2014	0	39	0.5%	1.5%	0.00 [0.00; 0.09]
Salim et al 2014	0	40	0.5%	1.5%	0.00 [0.00; 0.09]
Salim et al 2014	0	44	0.5%	1.5%	0.00 [0.00; 0.08]
Salim et al 2014	0	48	0.5%	1.5%	0.00 [0.00; 0.07]
Salim et al 2014	0	51	0.5%	1.5%	0.00 [0.00; 0.07]
Salim et al 2014	0	53	0.5%	1.5%	0.00 [0.00; 0.07]
Sharma et al 2001	0	5	0.4%	1.5%	0.00 [0.00; 0.52]
Sinshaw et al 2006	0	141	0.5%	1.5%	0.00 [0.00; 0.03]
Sinshaw et al 2006	0	608	0.5%	1.5%	0.00 [0.00; 0.01]
**Total (fixed effect, 95% CI)**		**3902**	**100.0%**	**--**	**0.17 [0.14; 0.20]**
**Total (random effects, 95% CI)**			**--**	**100.0%**	**0.04 [0.02; 0.07]**

Heterogeneity: $Tau^2 = 5.3639$; $Chi^2 = 559.72$, df = 62 ($P < 0.01$); $I^2 = 89\%$

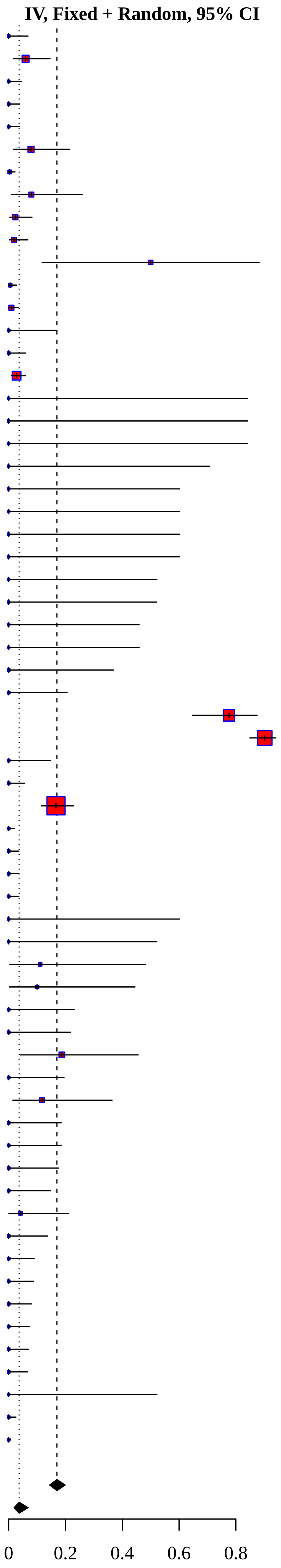

Pig

Study	Events	Total	Weight (fixed)	Weight (random)	IV, Fixed + Random, 95% CI
Balyeidhusa et al 2012	6	13	1.9%	6.0%	0.46 [0.19; 0.75]
Biryomumaisho et al 2009	8	253	4.6%	6.6%	0.03 [0.01; 0.06]
Biryomumaisho et al 2009	3	253	1.8%	5.9%	0.01 [0.00; 0.03]
Biryomumaisho et al 2013	1	253	0.6%	4.3%	0.00 [0.00; 0.02]
Hamill et al 2013	1	19	0.6%	4.3%	0.05 [0.00; 0.26]
Hamill et al 2013	1	42	0.6%	4.3%	0.02 [0.00; 0.13]
Hamill et al 2013	4	70	2.2%	6.1%	0.06 [0.02; 0.14]
Magona et al 1999	0	4	0.3%	2.9%	0.00 [0.00; 0.60]
Magona et al 1999	0	12	0.3%	3.1%	0.00 [0.00; 0.26]
Makumyaviri et al 1989	0	91	0.3%	3.1%	0.00 [0.00; 0.04]
Makumyaviri et al 1989	0	115	0.3%	3.1%	0.00 [0.00; 0.03]
N'Djetchi et al 2017	0	137	0.3%	3.1%	0.00 [0.00; 0.03]
Nakayima et al 2012	47	248	22.7%	7.1%	0.19 [0.14; 0.24]
Ng'ayo et al 2005	0	3	0.3%	2.9%	0.00 [0.00; 0.71]
Ng'ayo et al 2005	1	6	0.5%	4.0%	0.17 [0.00; 0.64]
Ng'ayo et al 2005	1	9	0.5%	4.1%	0.11 [0.00; 0.48]
Ng'ayo et al 2005	3	13	1.4%	5.6%	0.23 [0.05; 0.54]
Ng'ayo et al 2005	0	21	0.3%	3.1%	0.00 [0.00; 0.16]
Nimpaye et al 2011	111	307	42.2%	7.1%	0.36 [0.31; 0.42]
Simo et al 2006	46	133	17.9%	7.0%	0.35 [0.27; 0.43]
Simukoko et al 2007	0	324	0.3%	3.1%	0.00 [0.00; 0.01]
Simukoko et al 2007	0	324	0.3%	3.1%	0.00 [0.00; 0.01]
**Total (fixed effect, 95% CI)**		**2650**	**100.0%**	--	**0.23 [0.20; 0.26]**
**Total (random effects, 95% CI)**			--	**100.0%**	**0.06 [0.03; 0.10]**

Heterogeneity: $Tau^2 = 1.5394$; $Chi^2 = 211.16$, df = 21 ($P < 0.01$); $I^2 = 90\%$

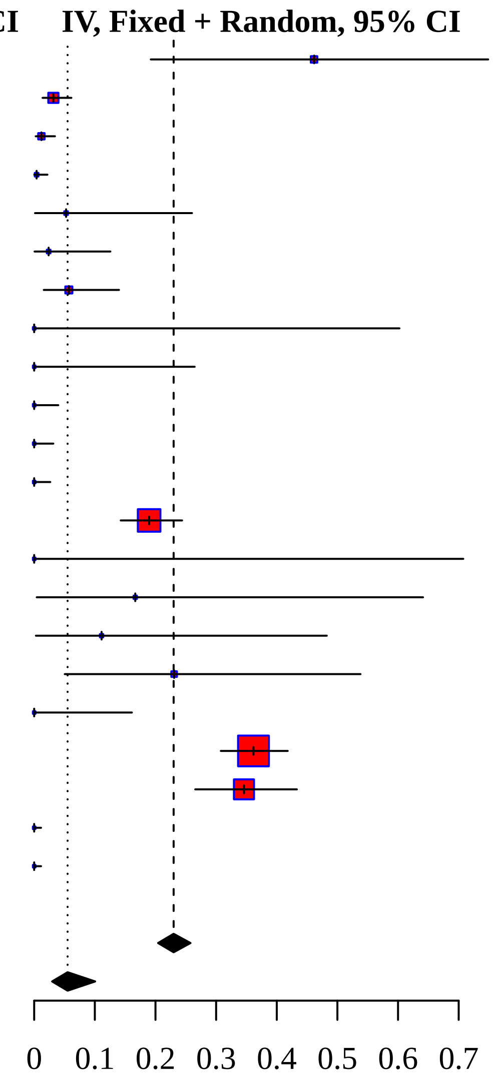

Small ruminant

Study	Events	Total	Weight (fixed)	Weight (random)	IV, Fixed + Random, 95% CI
Applewhaite 1990	1	77	0.2%	1.2%	0.01 [0.00; 0.07]
Applewhaite 1990	3	197	0.7%	1.5%	0.02 [0.00; 0.04]
Ávila Pulgarín et al 2013	0	11	0.1%	0.9%	0.00 [0.00; 0.28]
Ávila Pulgarín et al 2013	0	84	0.1%	1.0%	0.00 [0.00; 0.04]
Balyeidhusa et al 2012	5	13	0.7%	1.5%	0.38 [0.14; 0.68]
Balyeidhusa et al 2012	3	21	0.6%	1.5%	0.14 [0.03; 0.36]
Behnke et al 2011	2	1054	0.5%	1.4%	0.00 [0.00; 0.01]
Birhanu et al 2015	2	60	0.5%	1.4%	0.03 [0.00; 0.12]
Birhanu et al 2015	1	64	0.2%	1.2%	0.02 [0.00; 0.08]
Birhanu et al 2015	3	117	0.7%	1.5%	0.03 [0.01; 0.07]
Birhanu et al 2015	3	204	0.7%	1.5%	0.01 [0.00; 0.04]
Biryomumaisho et al 2013	1	573	0.2%	1.2%	0.00 [0.00; 0.01]
Cordon−Obras et al 2009	6	103	1.3%	1.6%	0.06 [0.02; 0.12]
Cordon−Obras et al 2009	2	228	0.5%	1.4%	0.01 [0.00; 0.03]
Cordon−Obras et al 2009	2	228	0.5%	1.4%	0.01 [0.00; 0.03]
Daniel et al 1994	22	615	4.9%	1.7%	0.04 [0.02; 0.05]
Dinka and Abebe 2005	4	196	0.9%	1.6%	0.02 [0.01; 0.05]
Dinka and Abebe 2005	3	337	0.7%	1.5%	0.01 [0.00; 0.03]
Garcia et al 2005	2	5	0.3%	1.3%	0.40 [0.05; 0.85]
Garcia et al 2005	5	5	0.1%	0.9%	1.00 [0.48; 1.00]
Garcia et al 2005	5	5	0.1%	0.9%	1.00 [0.48; 1.00]
Idehen et al 2018	6	101	1.3%	1.6%	0.06 [0.02; 0.12]
Joshua 1989	6	286	1.4%	1.6%	0.02 [0.01; 0.05]
Joshua 1989	5	221	1.1%	1.6%	0.02 [0.01; 0.05]
Kalu 1996	59	304	11.1%	1.7%	0.19 [0.15; 0.24]
Kalu 1996	15	239	3.3%	1.7%	0.06 [0.04; 0.10]
Kayang et al 1997	13	79	2.5%	1.7%	0.16 [0.09; 0.26]
Kayang et al 1997	14	131	2.9%	1.7%	0.11 [0.06; 0.17]
Kebede et al 2009	9	600	2.1%	1.6%	0.02 [0.01; 0.03]
Kebede et al 2009	49	1810	11.1%	1.7%	0.03 [0.02; 0.04]
Laohasinnarong et al 2015	2	36	0.4%	1.4%	0.06 [0.01; 0.19]
Magona et al 1999	0	4	0.1%	0.9%	0.00 [0.00; 0.60]
Magona et al 1999	0	12	0.1%	0.9%	0.00 [0.00; 0.26]
Magona et al 1999	2	14	0.4%	1.4%	0.14 [0.02; 0.43]
Magona et al 1999	0	27	0.1%	0.9%	0.00 [0.00; 0.13]
Magona et al 1999	0	75	0.1%	1.0%	0.00 [0.00; 0.05]
Makumyaviri et al 1989	0	57	0.1%	1.0%	0.00 [0.00; 0.06]
Makumyaviri et al 1989	1	58	0.2%	1.2%	0.02 [0.00; 0.09]
Makumyaviri et al 1989	0	58	0.1%	1.0%	0.00 [0.00; 0.06]
Makumyaviri et al 1989	1	91	0.2%	1.2%	0.01 [0.00; 0.06]
Mekonnen et al 2014	11	435	2.5%	1.7%	0.03 [0.01; 0.04]
Musinguzi et al 2016	20	53	2.9%	1.7%	0.38 [0.25; 0.52]
N'Djetchi et al 2017	1	136	0.2%	1.2%	0.01 [0.00; 0.04]
N'Djetchi et al 2017	9	192	2.0%	1.6%	0.05 [0.02; 0.09]
Ng'ayo et al 2005	0	7	0.1%	0.9%	0.00 [0.00; 0.41]
Ng'ayo et al 2005	0	8	0.1%	0.9%	0.00 [0.00; 0.37]
Ng'ayo et al 2005	1	13	0.2%	1.2%	0.08 [0.00; 0.36]
Ng'ayo et al 2005	0	30	0.1%	0.9%	0.00 [0.00; 0.12]
Ng'ayo et al 2005	4	35	0.8%	1.5%	0.11 [0.03; 0.27]
Ng'ayo et al 2005	0	37	0.1%	1.0%	0.00 [0.00; 0.09]
Ng'ayo et al 2005	0	37	0.1%	1.0%	0.00 [0.00; 0.09]
Ng'ayo et al 2005	0	42	0.1%	1.0%	0.00 [0.00; 0.08]
Ng'ayo et al 2005	5	55	1.1%	1.6%	0.09 [0.03; 0.20]
Ng'ayo et al 2005	8	86	1.7%	1.6%	0.09 [0.04; 0.18]
Nimpaye et al 2011	48	264	9.2%	1.7%	0.18 [0.14; 0.23]
Nimpaye et al 2011	23	267	4.9%	1.7%	0.09 [0.06; 0.13]
Nyimba et al 2015	0	183	0.1%	1.0%	0.00 [0.00; 0.02]
Nyimba et al 2015	8	239	1.8%	1.6%	0.03 [0.01; 0.06]
Pereira De Almeid et al 1998	3	27	0.6%	1.5%	0.11 [0.02; 0.29]
Pereira De Almeid et al 1998	17	27	1.5%	1.6%	0.63 [0.42; 0.81]
Pereira De Almeid et al 1998	1	49	0.2%	1.2%	0.02 [0.00; 0.11]
Pereira De Almeid et al 1998	0	49	0.1%	1.0%	0.00 [0.00; 0.07]
Ravel et al 2015	0	43	0.1%	1.0%	0.00 [0.00; 0.08]
Ravel et al 2015	1	52	0.2%	1.2%	0.02 [0.00; 0.10]
Robson and Rickman 1972	6	133	1.3%	1.6%	0.05 [0.02; 0.10]
Robson and Rickman 1972	2	133	0.5%	1.4%	0.02 [0.00; 0.05]
Samdi et al 2008	2	209	0.5%	1.4%	0.01 [0.00; 0.03]
Samdi et al 2008	3	320	0.7%	1.5%	0.01 [0.00; 0.03]
Sanni et al 2013	147	205	9.7%	1.7%	0.72 [0.65; 0.78]
Sharma et al 2001	0	10	0.1%	0.9%	0.00 [0.00; 0.31]
Simukoko et al 2007	0	333	0.1%	1.0%	0.00 [0.00; 0.01]
Simukoko et al 2007	0	333	0.1%	1.0%	0.00 [0.00; 0.01]
Sinshaw et al 2006	1	122	0.2%	1.2%	0.01 [0.00; 0.04]
Sinshaw et al 2006	1	676	0.2%	1.2%	0.00 [0.00; 0.01]
Vokaty et al1993	11	193	2.4%	1.7%	0.06 [0.03; 0.10]
**Total (fixed effect, 95% CI)**		**13433**	**100.0%**	−−	**0.09 [0.08; 0.10]**
**Total (random effects, 95% CI)**			−−	**100.0%**	**0.04 [0.03; 0.06]**

Heterogeneity: $Tau^2 = 2.5116$; $Chi^2 = 1092.05$, df = 74 ($P < 0.01$); $I^2 = 93\%$

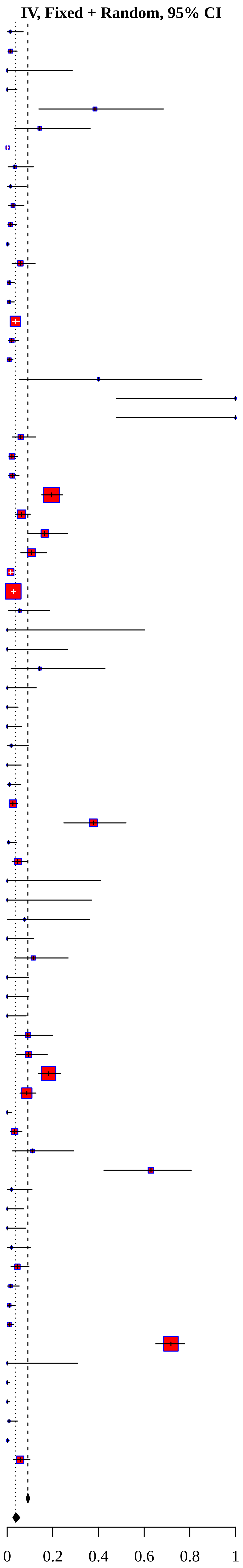

water buffalo

Study	Events	Total	Weight (fixed)	Weight (random)	IV, Fixed + Random, 95% CI
Garcia et al 2005	36	316	12.7%	19.8%	0.11 [0.08; 0.15]
Garcia et al 2005	121	316	29.6%	20.1%	0.38 [0.33; 0.44]
Garcia et al 2005	60	316	19.3%	20.0%	0.19 [0.15; 0.24]
Garcia et al 2016	67	293	20.5%	20.0%	0.23 [0.18; 0.28]
Tamasaukas et al 2006	180	240	17.9%	20.0%	0.75 [0.69; 0.80]
**Total (fixed effect, 95% CI)**		**1481**	**100.0%**	**--**	**0.32 [0.30; 0.35]**
**Total (random effects, 95% CI)**			**--**	**100.0%**	**0.31 [0.14; 0.54]**

Heterogeneity: $Tau^2 = 1.2364$; $Chi^2 = 248.38$, df = 4 ($P < 0.01$); $I^2 = 98\%$

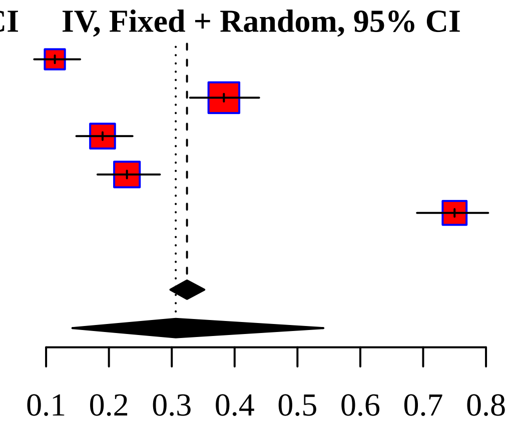

Wild animals

Study	Events	Total	Weight (fixed)	Weight (random)	IV, Fixed + Random, 95% CI
Anderson et al 2011	1	1	0.1%	0.6%	1.00 [0.03; 1.00]
Anderson et al 2011	5	10	0.7%	1.4%	0.50 [0.19; 0.81]
Anderson et al 2011	1	29	0.3%	1.0%	0.03 [0.00; 0.18]
Anderson et al 2011	1	56	0.3%	1.0%	0.02 [0.00; 0.10]
Anderson et al 2011	5	65	1.3%	1.6%	0.08 [0.03; 0.17]
Anderson et al 2011	0	3	0.1%	0.6%	0.00 [0.00; 0.71]
Anderson et al 2011	0	28	0.1%	0.7%	0.00 [0.00; 0.12]
Anderson et al 2011	0	5	0.1%	0.7%	0.00 [0.00; 0.52]
Anderson et al 2011	0	2	0.1%	0.6%	0.00 [0.00; 0.84]
Anderson et al 2011	0	1	0.1%	0.6%	0.00 [0.00; 0.98]
Anderson et al 2011	0	7	0.1%	0.7%	0.00 [0.00; 0.41]
Anderson et al 2011	0	1	0.1%	0.6%	0.00 [0.00; 0.98]
Anderson et al 2011	0	20	0.1%	0.7%	0.00 [0.00; 0.17]
Anderson et al 2011	0	4	0.1%	0.7%	0.00 [0.00; 0.60]
Anderson et al 2011	0	4	0.1%	0.7%	0.00 [0.00; 0.60]
Anderson et al 2011	0	7	0.1%	0.7%	0.00 [0.00; 0.41]
Anderson et al 2011	0	47	0.1%	0.7%	0.00 [0.00; 0.08]
Anderson et al 2011	0	14	0.1%	0.7%	0.00 [0.00; 0.23]
Anderson et al 2011	0	14	0.1%	0.7%	0.00 [0.00; 0.23]
Anderson et al 2011	0	57	0.1%	0.7%	0.00 [0.00; 0.06]
Anderson et al 2011	0	5	0.1%	0.7%	0.00 [0.00; 0.52]
Anderson et al 2011	0	1	0.1%	0.6%	0.00 [0.00; 0.98]
Anderson et al 2011	0	10	0.1%	0.7%	0.00 [0.00; 0.31]
Anderson et al 2011	0	27	0.1%	0.7%	0.00 [0.00; 0.13]
Dillmann & Townsend 1979	1	19	0.3%	1.0%	0.05 [0.00; 0.26]
Dillmann & Townsend 1979	4	23	0.9%	1.5%	0.17 [0.05; 0.39]
Dillmann & Townsend 1979	0	1	0.1%	0.6%	0.00 [0.00; 0.98]
Dillmann & Townsend 1979	0	6	0.1%	0.7%	0.00 [0.00; 0.46]
Dillmann & Townsend 1979	0	1	0.1%	0.6%	0.00 [0.00; 0.98]
Dillmann & Townsend 1979	1	7	0.2%	1.0%	0.14 [0.00; 0.58]
Dillmann & Townsend 1979	1	3	0.2%	0.8%	0.33 [0.01; 0.91]
Dillmann & Townsend 1979	0	20	0.1%	0.7%	0.00 [0.00; 0.17]
Dillmann & Townsend 1979	0	6	0.1%	0.7%	0.00 [0.00; 0.46]
Dillmann & Townsend 1979	0	1	0.1%	0.6%	0.00 [0.00; 0.98]
Dillmann & Townsend 1979	0	5	0.1%	0.7%	0.00 [0.00; 0.52]
Dillmann & Townsend 1979	0	10	0.1%	0.7%	0.00 [0.00; 0.31]
Dillmann & Townsend 1979	0	250	0.1%	0.7%	0.00 [0.00; 0.01]
Dillmann & Townsend 1979	0	7	0.1%	0.7%	0.00 [0.00; 0.41]
Dillmann & Townsend 1979	0	23	0.1%	0.7%	0.00 [0.00; 0.15]
Dillmann & Townsend 1979	0	1	0.1%	0.6%	0.00 [0.00; 0.98]
Dillmann & Townsend 1979	7	13	0.9%	1.5%	0.54 [0.25; 0.81]
Dillmann & Townsend 1979	0	2	0.1%	0.6%	0.00 [0.00; 0.84]
Dillmann & Townsend 1979	0	6	0.1%	0.7%	0.00 [0.00; 0.46]
Dillmann & Townsend 1979	0	2	0.1%	0.6%	0.00 [0.00; 0.84]
Dillmann & Townsend 1979	0	18	0.1%	0.7%	0.00 [0.00; 0.19]
Dillmann & Townsend 1979	1	24	0.3%	1.0%	0.04 [0.00; 0.21]
Dillmann & Townsend 1979	0	1	0.1%	0.6%	0.00 [0.00; 0.98]
Dillmann & Townsend 1979	0	5	0.1%	0.7%	0.00 [0.00; 0.52]
Dillmann & Townsend 1979	0	11	0.1%	0.7%	0.00 [0.00; 0.28]
Dillmann & Townsend 1979	0	2	0.1%	0.6%	0.00 [0.00; 0.84]
Dillmann & Townsend 1979	0	24	0.1%	0.7%	0.00 [0.00; 0.14]
Dillmann & Townsend 1979	14	20	1.1%	1.6%	0.70 [0.46; 0.88]
Dillmann & Townsend 1979	0	1	0.1%	0.6%	0.00 [0.00; 0.98]
Dillmann & Townsend 1979	0	2	0.1%	0.6%	0.00 [0.00; 0.84]
Dillmann & Townsend 1979	0	5	0.1%	0.7%	0.00 [0.00; 0.52]
Dillmann & Townsend 1979	0	5	0.1%	0.7%	0.00 [0.00; 0.52]
Dillmann & Townsend 1979	0	20	0.1%	0.7%	0.00 [0.00; 0.17]
Dillmann & Townsend 1979	0	2	0.1%	0.6%	0.00 [0.00; 0.84]
Drager and Mehlitz 1978	2	2	0.1%	0.6%	1.00 [0.16; 1.00]
Drager and Mehlitz 1978	6	15	1.0%	1.5%	0.40 [0.16; 0.68]
Drager and Mehlitz 1978	7	22	1.3%	1.6%	0.32 [0.14; 0.55]
Drager and Mehlitz 1978	19	23	0.9%	1.5%	0.83 [0.61; 0.95]
Drager and Mehlitz 1978	14	23	1.5%	1.6%	0.61 [0.39; 0.80]
Drager and Mehlitz 1978	10	50	2.2%	1.7%	0.20 [0.10; 0.34]
Drager and Mehlitz 1978	29	60	4.1%	1.8%	0.48 [0.35; 0.62]
Drager and Mehlitz 1978	36	416	9.0%	1.9%	0.09 [0.06; 0.12]
Drager and Mehlitz 1978	243	605	39.6%	1.9%	0.40 [0.36; 0.44]
Guedegbe et al 1992	1	20	0.3%	1.0%	0.05 [0.00; 0.25]
Guedegbe et al 1992	1	50	0.3%	1.0%	0.02 [0.00; 0.11]
Guedegbe et al 1992	0	3	0.1%	0.6%	0.00 [0.00; 0.71]
Guedegbe et al 1992	0	3	0.1%	0.6%	0.00 [0.00; 0.71]
Guedegbe et al 1992	0	3	0.1%	0.6%	0.00 [0.00; 0.71]
Njiokou et al 2004	3	37	0.8%	1.5%	0.08 [0.02; 0.22]
Njiokou et al 2004	24	290	6.0%	1.8%	0.08 [0.05; 0.12]
Njiokou et al 2004	2	12	0.5%	1.3%	0.17 [0.02; 0.48]
Njiokou et al 2004	0	2	0.1%	0.6%	0.00 [0.00; 0.84]
Njiokou et al 2004	1	3	0.2%	0.8%	0.33 [0.01; 0.91]
Njiokou et al 2004	1	1	0.1%	0.6%	1.00 [0.03; 1.00]
Njiokou et al 2004	5	55	1.2%	1.6%	0.09 [0.03; 0.20]
Njiokou et al 2004	22	155	5.1%	1.8%	0.14 [0.09; 0.21]
Njiokou et al 2004	2	14	0.5%	1.3%	0.14 [0.02; 0.43]
Njiokou et al 2004	1	8	0.2%	1.0%	0.12 [0.00; 0.53]
Njiokou et al 2004	0	5	0.1%	0.7%	0.00 [0.00; 0.52]
Njiokou et al 2004	8	46	1.8%	1.7%	0.17 [0.08; 0.31]
Njiokou et al 2004	11	101	2.7%	1.8%	0.11 [0.06; 0.19]
Njiokou et al 2004	0	1	0.1%	0.6%	0.00 [0.00; 0.98]
Njiokou et al 2004	0	5	0.1%	0.7%	0.00 [0.00; 0.52]
Njiokou et al 2004	0	3	0.1%	0.6%	0.00 [0.00; 0.71]
Njiokou et al 2004	0	5	0.1%	0.7%	0.00 [0.00; 0.52]
Njiokou et al 2004	1	5	0.2%	0.9%	0.20 [0.01; 0.72]
Njiokou et al 2004	0	1	0.1%	0.6%	0.00 [0.00; 0.98]
Njiokou et al 2004	2	5	0.3%	1.1%	0.40 [0.05; 0.85]
Njiokou et al 2004	0	3	0.1%	0.6%	0.00 [0.00; 0.71]
Njiokou et al 2004	3	8	0.5%	1.3%	0.38 [0.09; 0.76]
Njiokou et al 2004	7	106	1.8%	1.7%	0.07 [0.03; 0.13]
Njiokou et al 2004	0	15	0.1%	0.7%	0.00 [0.00; 0.22]
Njiokou et al 2004	0	1	0.1%	0.6%	0.00 [0.00; 0.98]
Njiokou et al 2004	4	135	1.1%	1.6%	0.03 [0.01; 0.07]
Njiokou et al 2004	0	1	0.1%	0.6%	0.00 [0.00; 0.98]
Njiokou et al 2004	0	1	0.1%	0.6%	0.00 [0.00; 0.98]
Njiokou et al 2004	0	6	0.1%	0.7%	0.00 [0.00; 0.46]
Njiokou et al 2004	0	8	0.1%	0.7%	0.00 [0.00; 0.37]
Njiokou et al 2004	1	8	0.2%	1.0%	0.12 [0.00; 0.53]
Njiokou et al 2004	0	3	0.1%	0.6%	0.00 [0.00; 0.71]
Njiokou et al 2004	3	32	0.7%	1.4%	0.09 [0.02; 0.25]
Njiokou et al 2004	2	34	0.5%	1.3%	0.06 [0.01; 0.20]
Njiokou et al 2004	5	20	1.0%	1.6%	0.25 [0.09; 0.49]
Njiokou et al 2004	0	5	0.1%	0.7%	0.00 [0.00; 0.52]
Silveira et al 2013	0	60	0.1%	0.7%	0.00 [0.00; 0.06]
**Total (fixed effect, 95% CI)**		**3459**	**100.0%**	--	**0.23 [0.21; 0.25]**
**Total (random effects, 95% CI)**			--	**100.0%**	**0.13 [0.10; 0.17]**

Heterogeneity: $Tau^2 = 1.1693$; $Chi^2 = 461.40$, df = 108 ($P < 0.01$); $I^2 = 77\%$

IV, Fixed + Random, 95% CI

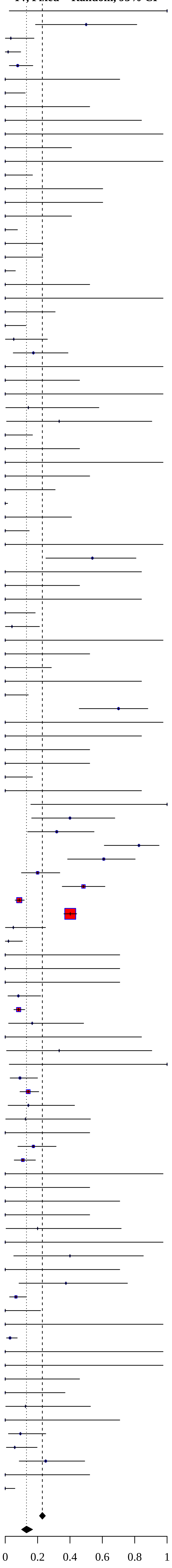

Supplement: Supplementary file 2 — Additional file 2. Forest plots showing an overview of studies reporting Trypanosoma vivax in different host species. [file 13071_2021_4584_MOESM2_ESM.pdf]
