# Supplementary figures and images for: Global distribution, host range and prevalence of Trypanosoma vivax: a systematic review and meta-analysis
Source: Parasit Vectors. 2021 Jan 25;14:80. doi: 10.1186/s13071-021-04584-x (PMC7830052; doi:10.1186/s13071-021-04584-x)

Cattle

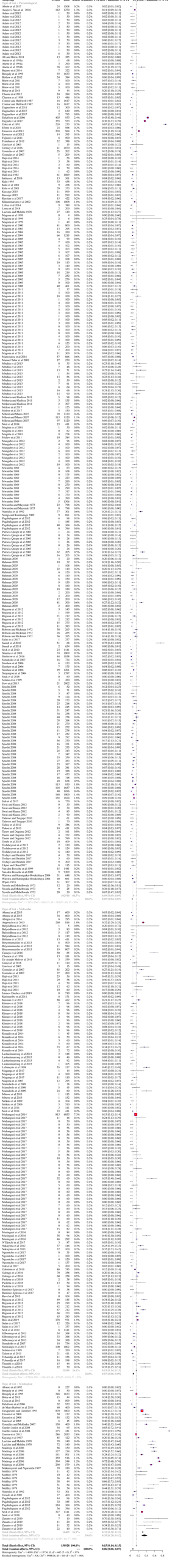

Dog

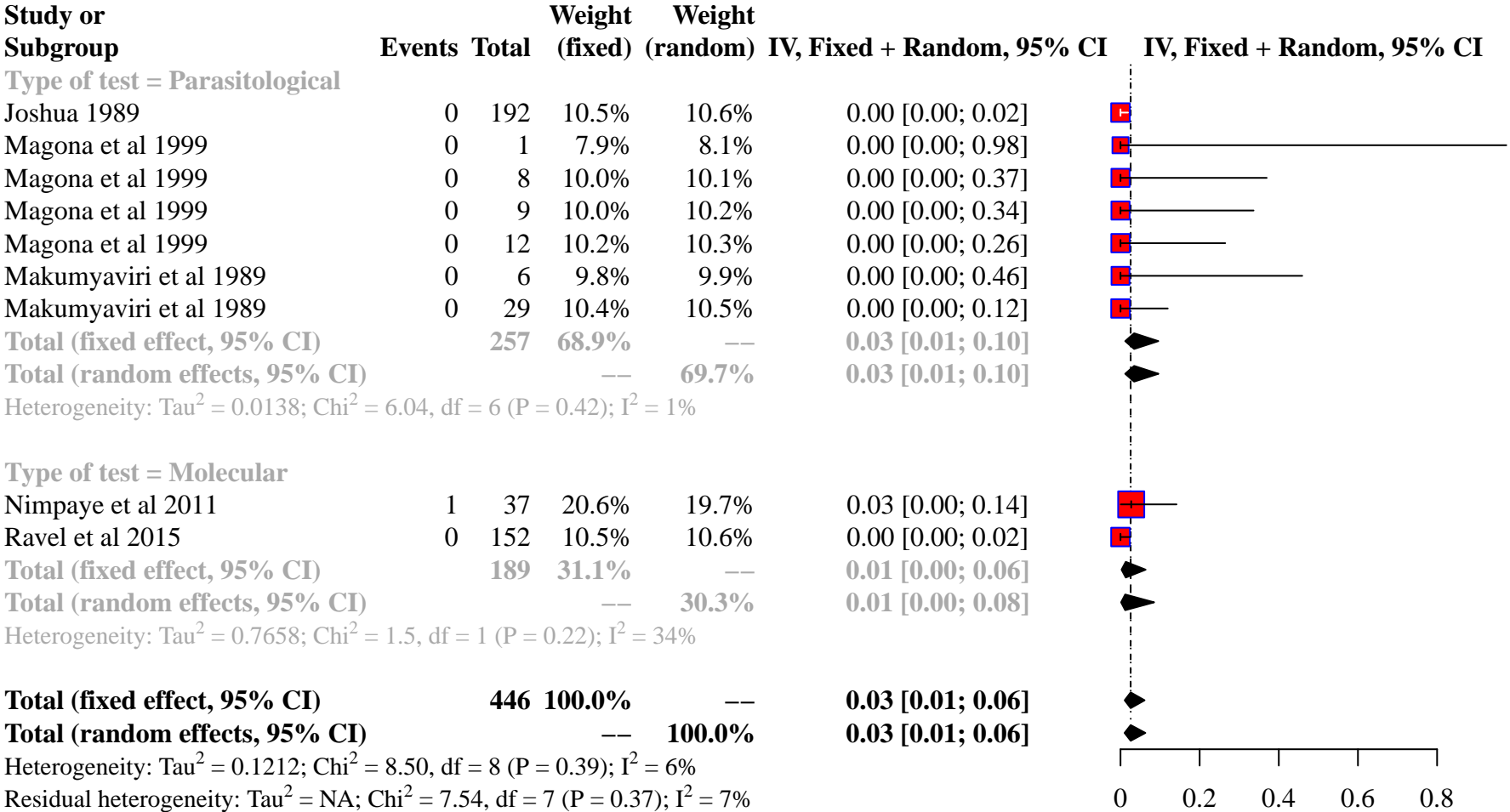

Equine

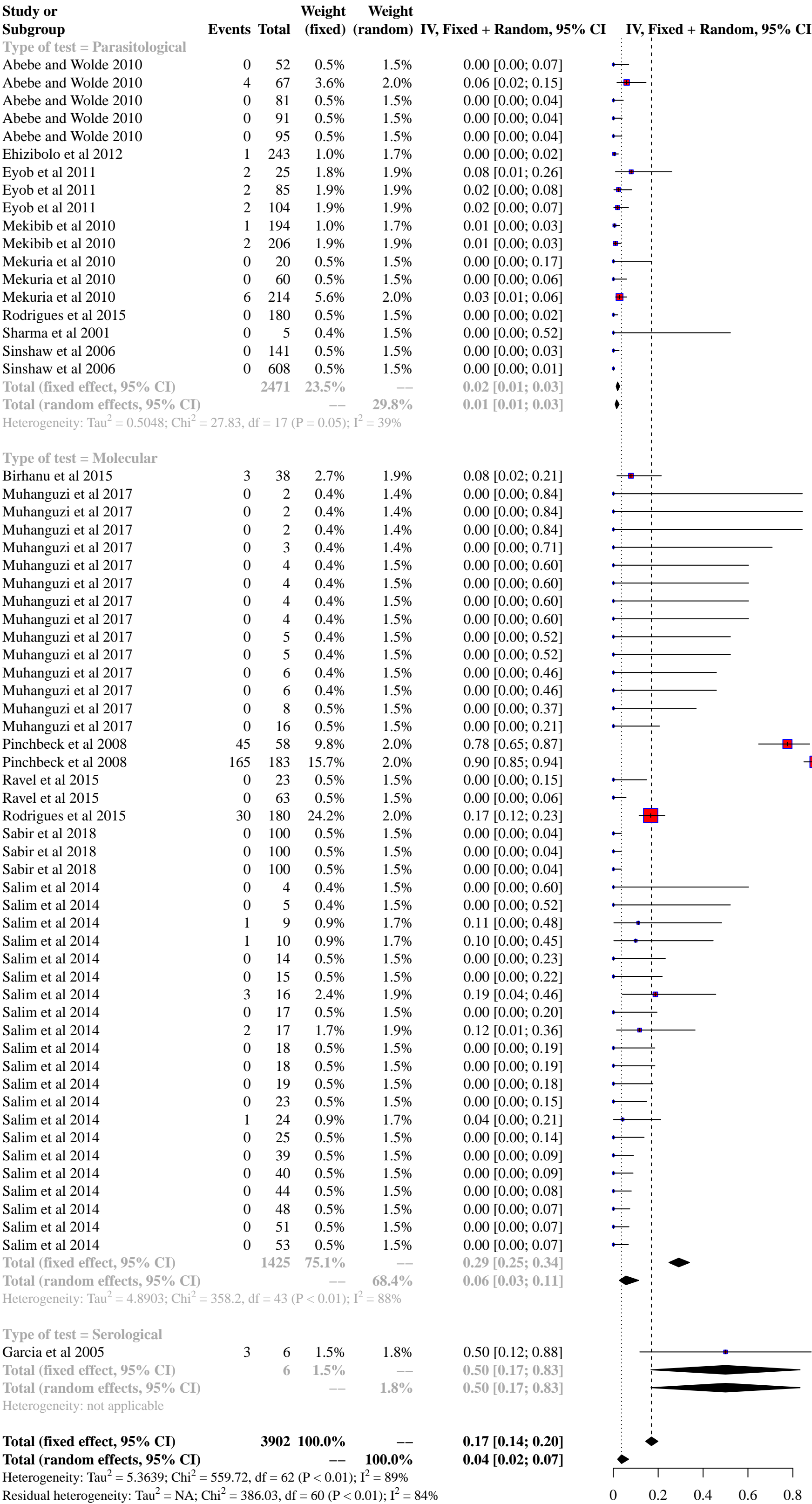

Pig

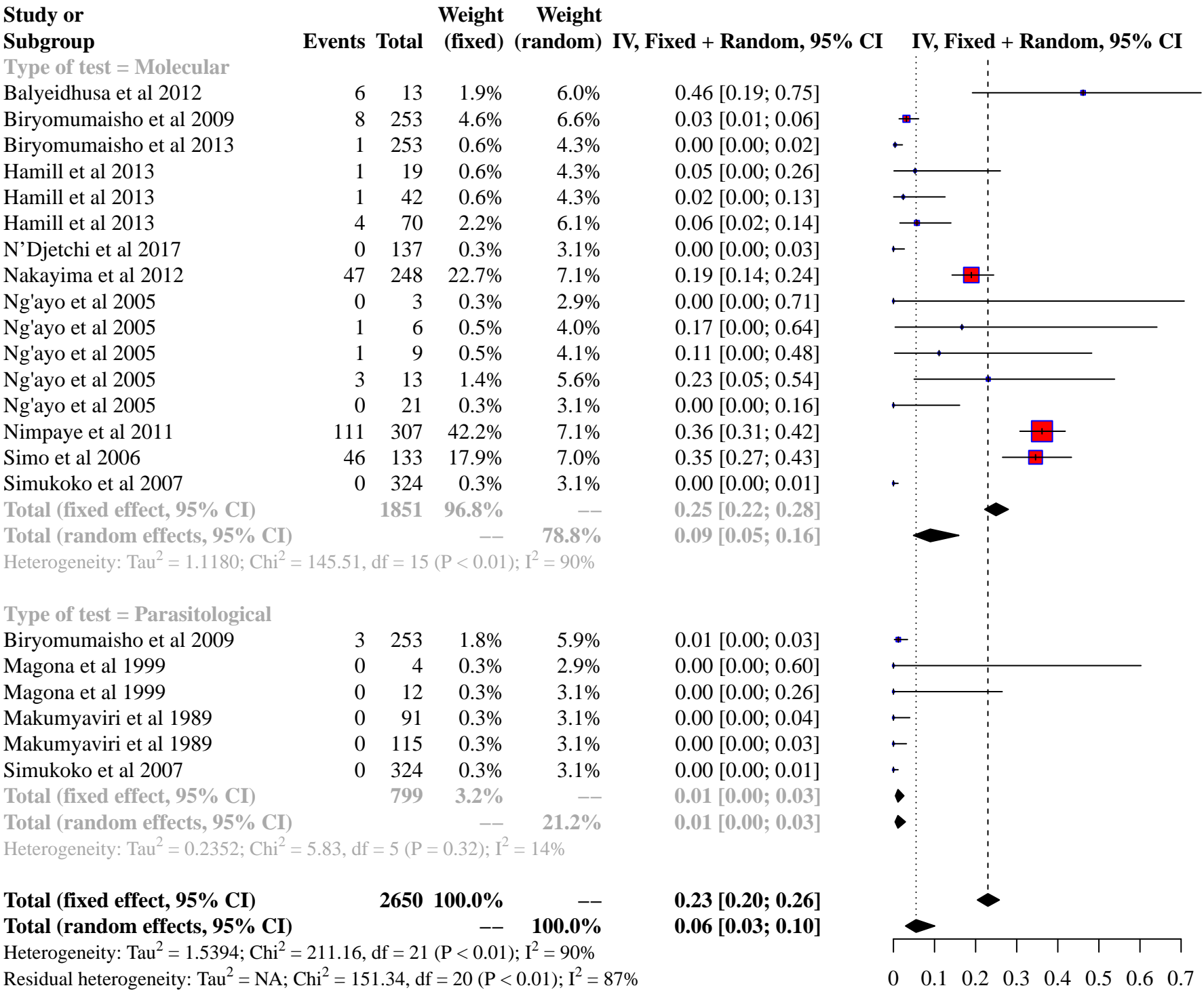

Small ruminant

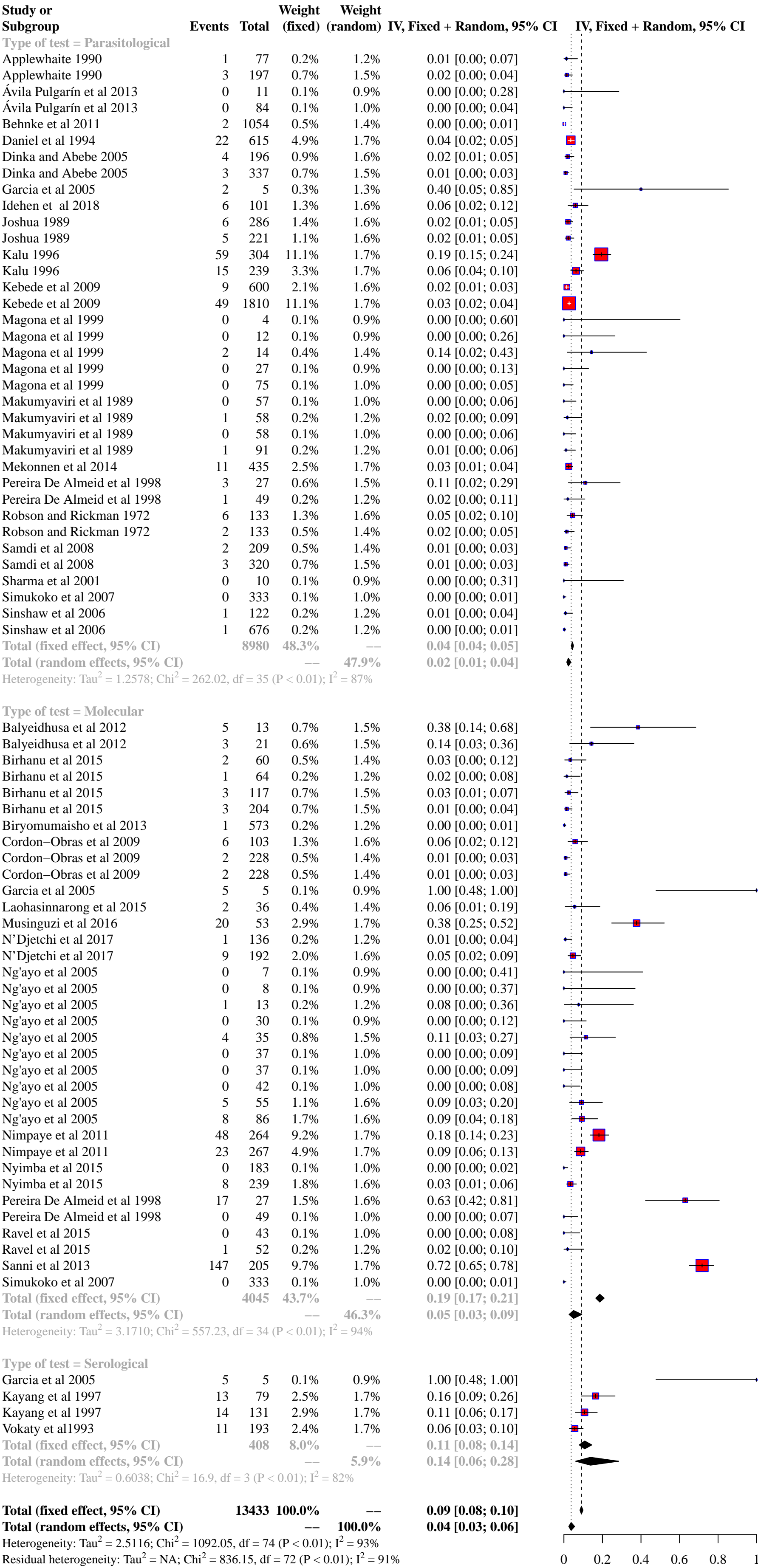

water buffalo

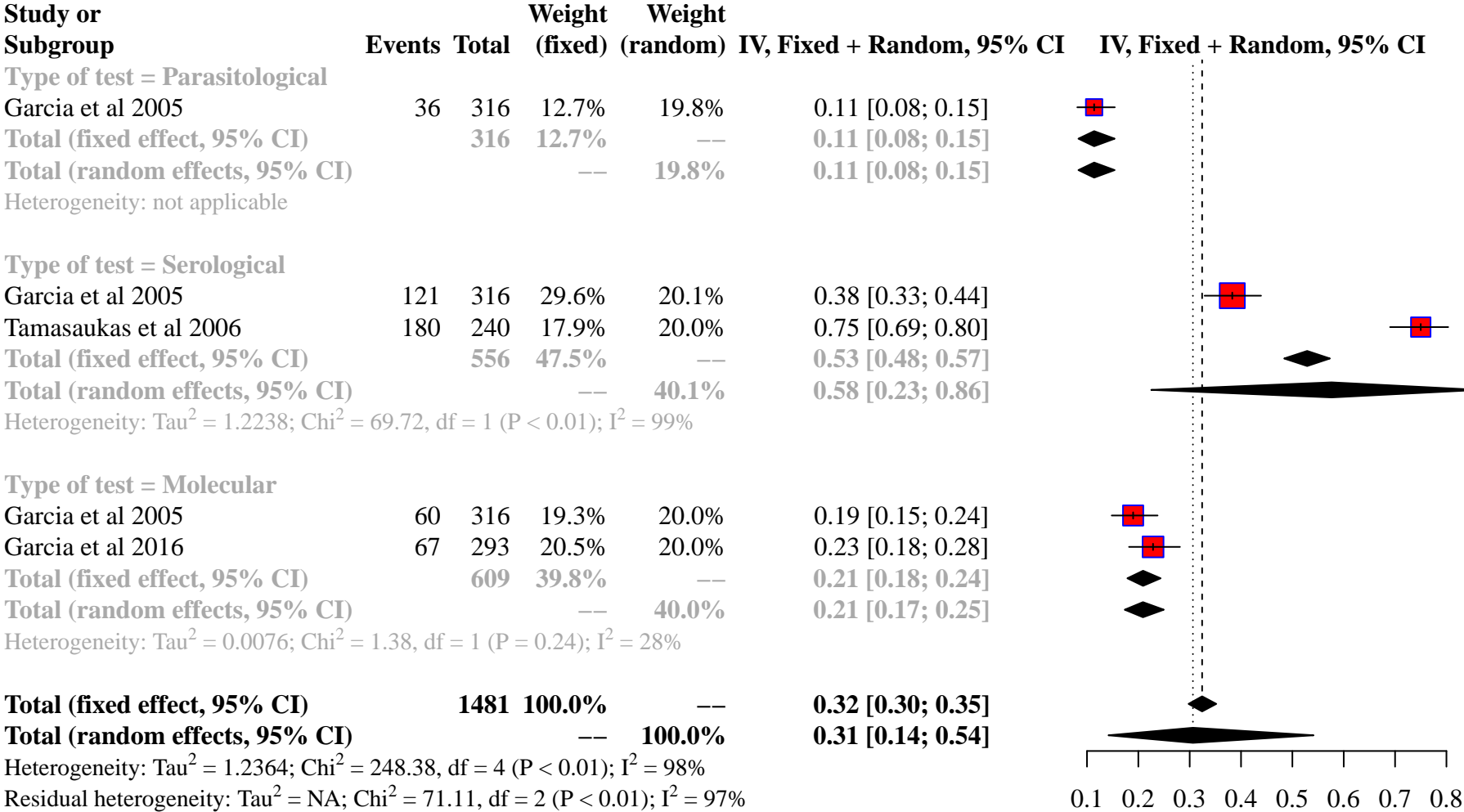

Wild animals

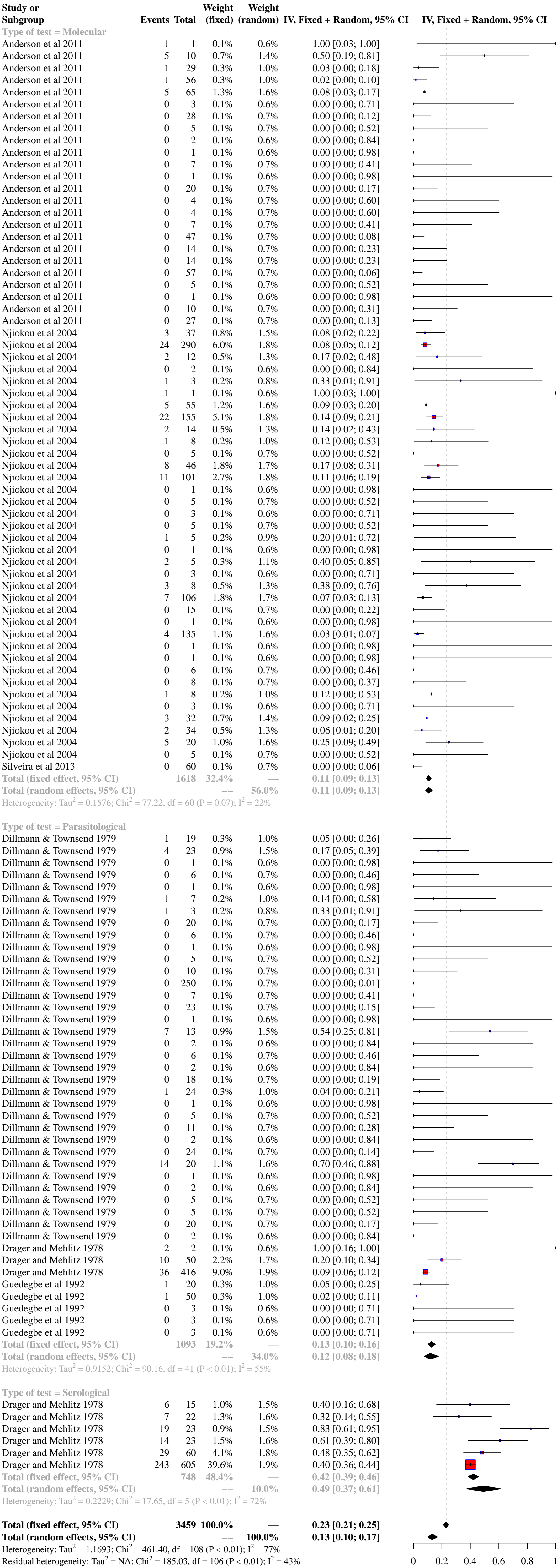

Supplement: Supplementary file 3 — Additional file 3. Forest plots showing an overview of studies reporting Trypanosoma vivax grouped by test methods in different host species. [file 13071_2021_4584_MOESM3_ESM.pdf]
